# Comparative efficacy of early exercise interventions for recovery outcomes after total knee arthroplasty: a systematic review and network meta-analysis

**DOI:** 10.3389/fmed.2026.1883684

**Published:** 2026-07-17

**Authors:** Xinyue Yan, PinMei Li, Jia Li, Liting Song, Jiapeng Jing, Xiaochen Fu, Zhuo Xu

**Affiliations:** 1 Department of Rehabilitation, China-Japan Union Hospital of Jilin University, Changchun, China; 2 Rehabilitation Therapeutics, School of Nursing, Jilin University, Changchun, China; 3 Department of Rehabilitation, The Second Hospital of Jilin University, Changchun, China

**Keywords:** early rehabilitation, exercise therapy, joint function, pain, range of motion, total knee arthroplasty

## Abstract

**Background:**

Early rehabilitation after total knee arthroplasty (TKA) is essential for pain relief and functional recovery. Although exercise is a core component of rehabilitation, the comparative effectiveness and optimal protocols of specific exercise modalities within the 3-day postoperative “golden period” remain uncertain. This network meta-analysis (NMA) compared the effects of different early exercise interventions (initiated ≤3 days post-TKA) on pain, joint function (WOMAC), and range of motion (ROM) and ranked their efficacy.

**Methods:**

Randomized controlled trials (RCTs) were systematically searched for using the PubMed, Embase, Cochrane Library, Web of Science, and Scopus databases for studies published in the past 10 years. Two reviewers independently screened the studies, extracted the data, and assessed the risk of bias using Cochrane Risk of Bias Tool (RoB2). A frequentist random-effects NMA was performed using Stata 18.0. Interventions were ranked using the surface under the cumulative ranking curve (SUCRA) values, and evidence certainty was evaluated using CINeMA.

**Results:**

A total of 32 RCTs (1,871 patients) evaluating nine interventions were included. In terms of pain relief, functional training and resistance training showed favorable comparative effects versus the control, with relatively high ranking probabilities (SMD: −4.23 and −1.57; SUCRA: 100 and 85.8%, respectively). For WOMAC-based functional status, functional training and intelligence-assisted training appeared to provide greater improvement than the other interventions in the current network (SMD: −1.51 and −1.35; SUCRA: 85.5 and 78.7%, respectively). Regarding ROM improvement, intelligence-assisted training and resistance training had the highest ranking probabilities and showed favorable effect estimates (SMD: 2.21 and 1.91; SUCRA: 94.7 and 88.9%, respectively). According to CINeMA, the certainty of evidence was moderate for pain and low for WOMAC and ROM.

**Conclusion:**

Functional training, intelligence-assisted training, and resistance training appeared to be promising early exercise strategies after TKA. Functional training showed favorable comparative effects on pain relief and functional improvement, while intelligence-assisted training demonstrated potential benefits across all three outcomes. To optimize patient recovery, clinicians should tailor the selection and combination of these interventions on the basis of the rehabilitation stage and goals.

**Systematic review registration:**

PROSPERO (CRD420251041523).

## Introduction

Knee osteoarthritis (KOA) is a major global health concern strongly linked to aging, and it is characterized by the failure of joint repair due to articular or periarticular abnormalities such as meniscal tears or synovial hyperplasia (1, 2). According to the 2021 Global Burden of Disease (GBD) report, approximately 375 million people exhibit KOA worldwide, with a higher incidence in women and adults aged >50 years (3). With population aging, diagnostic and treatment demands are increasing, resulting in higher rates for total knee arthroplasty (TKA) in the future (4). As the gold-standard intervention for advanced KOA, TKA can successfully alleviate pain, restore function, and enhance quality of life (5, 6). However, significant surgical trauma can result in persistent postoperative pain, functional limitations, and impaired proprioception (7, 8), as well as muscle weakness and long-term deficits (9–11). Consequently, early rehabilitation is vital for optimizing surgical outcomes.

The adoption of enhanced recovery after surgery (ERAS) protocols has markedly reduced the hospital length of stay (LOS) after TKA (12). For patients undergoing TKA, the average postoperative LOS is approximately 3 days (13, 14), underscoring the need for more timely and tailored rehabilitation interventions. The current evidence indicates that initiating rehabilitation within 3 days after surgery, especially within the 24-h window, can significantly reduce the length of hospital stay, costs, and the rate of complications such as deep vein thrombosis and pulmonary infection, confirming the clinical and economic value of early rehabilitation (15, 16).

Exercise training is a core component of post-TKA rehabilitation, and it is essential for pain relief, improvement in joint range of motion (ROM), and functional recovery. The exercises implemented vary widely and include active exercises such as resistance training, passive exercises such as continuous passive motion (CPM), intelligence-assisted training (e.g., virtual reality), and integrated exercise protocols. In recent years, with the advances in artificial intelligence and digital technology, intelligence-assisted training has been increasingly utilized to improve rehabilitation outcomes (17–19). However, existing randomized controlled trials and conventional pairwise meta-analyses have reported inconsistent findings on the comparative efficacy of early exercise interventions. For instance, one meta-analysis showed that lower-limb resistance training improved short-term pain and knee flexion range of motion but had limited effects on walking ability (20). In contrast, some randomized controlled trials reported that just a few in-hospital sessions of high-intensity resistance training significantly improved pain and physical function (21). For intelligence-assisted training such as virtual reality, some studies found it superior to conventional rehabilitation (22), while others reported no statistically significant differences (23). This heterogeneity in effect estimates suggests that pairwise comparisons alone cannot reliably determine which early exercise intervention is optimal. Nevertheless, updated systematic comparisons of the relative effectiveness and optimal protocols of different exercise modalities implemented within the critical 3-day postoperative window are lacking.

Therefore, in this study, a network meta-analysis (NMA) was conducted to synthesize direct and indirect evidence from the past decade. This study also compared the effects of various early exercise interventions initiated within 3 days after TKA on functional recovery and pain relief, as well as ranked their efficacy. The results may provide evidence-based guidance for executing precise and tailored clinical rehabilitation.

## Materials and methods

This systematic review and NMA was conducted in accordance with the Preferred Reporting Items for Systematic Reviews and Meta-Analyses extension for Network Meta-Analyses (PRISMA-NMA) guidelines and the Cochrane Handbook. The study protocol was based on the PICOS framework and was registered at PROSPERO (CRD420251041523).

### Data sources and search strategy

A systematic literature search was performed using the five following databases: PubMed, Embase, Cochrane Central Register of Controlled Trials, Web of Science, and Scopus. The search period was from January 2015 to October 2025. As the search terms, we employed a combination of Medical Subject Headings (MeSH in PubMed, Emtree in Embase) and free-text terms related to “Arthroplasty, Replacement, Knee,” “rehabilitation,” “exercise,” and “Randomized Controlled Trial.” The search terms were combined using Boolean operators (“OR” within concepts, “AND” between concepts). The reference lists of the relevant reviews and included studies were screened manually, and Google Scholar was searched for additional records. The complete search strategy is available in Supplementary Information S2.

### Inclusion and exclusion criteria

Studies were included according to the PICOS criteria: (1) population: patients undergoing TKA, regardless of age, ethnicity, BMI, etc.; (2) intervention: any exercise rehabilitation program initiated within 3 days post-TKA, including active, passive, technology-assisted, or comprehensive training; (3) control: any alternative exercise program, standard care, or conventional rehabilitation (e.g., simple activities, manual therapy, or health education); (4) outcomes: studies reporting at least one of the following outcomes: pain, ROM, and Western Ontario and McMaster Universities Osteoarthritis Index (WOMAC); and (5) study design: only RCTs with no significant baseline differences and noncrossover designs. The exclusion criteria were as follows: (1) the study type not explicitly specified or non-RCT literature, such as surveys, descriptive studies, meta-analyses, systematic reviews, reviews, basic research, or research protocols; (2) duplicate studies or studies where the full text was unavailable; (3) studies involving patients undergoing revision surgery or partial knee arthroplasty (unicompartmental replacement); (4) studies with insufficient sample sizes (<10 participants); (5) studies in which the intervention was initiated later than 3 days post-surgery or at an unspecified time point; studies involving nonexercise physical therapy (e.g., ice application alone, heat application, electrotherapy); studies comparing only different doses of the same exercise modality (e.g., high-intensity vs. low-intensity training); and (6) studies with unclear data that could not be extracted or obtained from the authors.

### Study selection and data extraction

Following the removal of duplicate studies using Endnote, two researchers (XY and LT) independently screened the titles and abstracts against the eligibility criteria and excluded clearly irrelevant studies. A full-text review was conducted for potentially eligible articles. Any disagreements were resolved through discussion or consultation with a third researcher.

Using predesigned Excel templates, the two researchers independently collected the following information: general details (first author, publication year, country, study type, sample size), patient characteristics (gender distribution, age range), intervention details (group assignment, intervention method, start date, frequency, intensity, single session duration, intervention cycle), and outcome measures (pain scores, WOMAC, ROM). Baseline and postintervention data were extracted, and intention-to-treat results were prioritized in the analysis; otherwise, per-protocol data were utilized. In case of incomplete statistics reporting, estimates were derived following the guidelines in the Cochrane Handbook. Numeric data presented graphically were extracted using GetData Graph Digitizer (v2.20). Discrepancies in data extraction were resolved through consensus or consultation with a third researcher.

### Quality assessment and risk of bias

The risk of bias of the included studies was evaluated by at least two independent reviewers (XY and LT) using the revised Cochrane Risk of Bias tool (RoB2, 24). This tool assesses the following five domains: randomization, deviations from intended interventions, missing outcome data, outcome measurement, and selective reporting. Each study was judged to have a “low,” “some concerns,” or “high” overall risk of bias. Any disagreements were resolved through consensus or consultation with a third researcher.

### Certainty of evidence

The certainty of evidence from this NMA was assessed using the CINeMA online tool (25, 26); the Cochrane Collaboration recommends this tool for evaluating the results of each comparison in an NMA. The evidence for each outcome was graded as high, moderate, low, or very low. Initially, the certainty of evidence from RCTs can be rated as high but can be downgraded on the basis of the following six domains: risk of bias, publication bias, indirectness, imprecision, heterogeneity, and inconsistency. Any differences in the evaluations were resolved by a third researcher.

### Data analyses

A frequentist NMA was performed using Stata 18.0 (27). Initially, pairwise meta-analyses of all outcomes were conducted using a random-effects model, in which each exercise intervention was compared against the control. Effect sizes were expressed as standardized mean differences (SMDs) with 95% confidence intervals (CIs) based on postintervention scores. Heterogeneity was quantified using the *I*^2^ statistic, with the values of 25, 50, and 75% denoting low, moderate, and high heterogeneity, respectively.

NMA was subsequently performed to integrate the direct and indirect evidence within a unified model, enabling the comparison and ranking of all the interventions (28). A network plot was generated, where the nodes represent interventions (weighted by the participant number) and the connecting lines represent direct comparisons (weighted by the number of trials) (27). To ensure consistency, clinical and methodological characteristics across studies were evaluated prior to synthesis. A random-effects model was used to account for between-study heterogeneity and provide more conservative CIs (29). Global inconsistency was assessed using a design-by-treatment interaction model (30), whereas local inconsistency was evaluated using the node-splitting method.

For clinical interpretation, SMDs were categorized as small (<0.40), moderate (0.40–0.80), or large (>0.80) according to the Cochrane guidelines. Interventions were ranked using the surface under the cumulative ranking curve (SUCRA) values and mean ranks, with higher SUCRA values (ranging from 0 to 100%) indicating a greater probability of being ranked favorably within the network (31). Potential publication bias was assessed using funnel plots.

## Results

### Study selection

**Figure 1:**
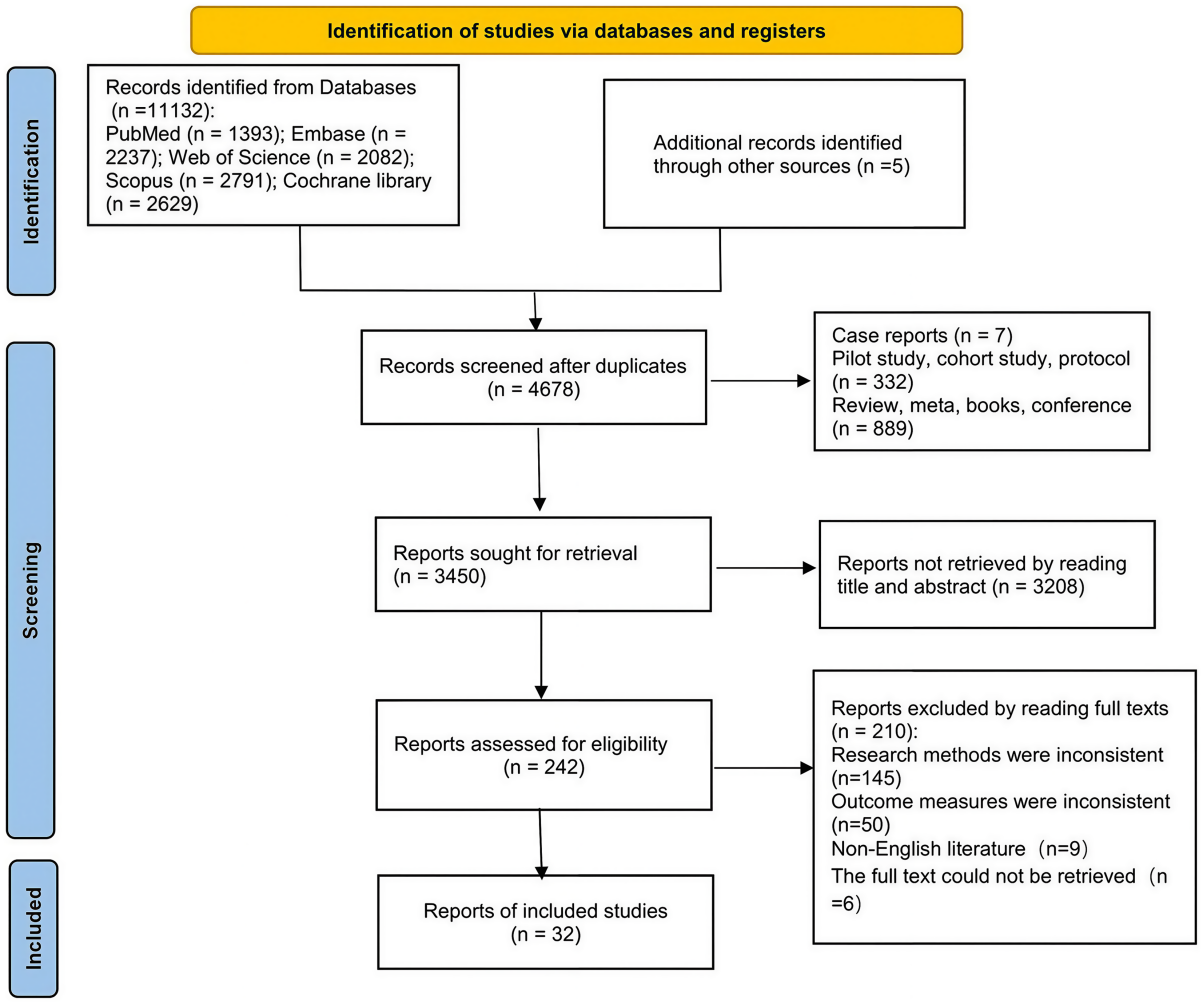
Flowchart of the study selection process for included studies.

The initial database search identified 11,132 records, supplemented by 5 records from manual screening. After the removal of 6,459 duplicates, 4,678 records remained. Among these, 1,228 studies were excluded because they were case reports (*n* = 7), pilot/cohort studies or protocols (*n* = 332), or reviews/meta-analyses/conference papers (*n* = 889). The titles and abstracts of the remaining 3,450 studies were screened, and 3,208 irrelevant studies were excluded. The full texts of 242 potentially eligible articles were assessed, resulting in the exclusion of 210 articles because of irrelevant research content (*n* = 145), mismatched outcome measures (*n* = 50), non-English language (*n* = 9), or unavailability of the full text (*n* = 6). Finally, 32 studies were included in the analysis. The literature screening process is summarized in Figure 1.

### Characteristics of included studies

A total of 32 RCTs (21, 32–62)(published from 2015 to 2025) involving 1,871 patients (intervention: *n* = 953; control: *n* = 918) who underwent TKA were included. The studies were conducted across 11 countries, including China, the United States, Japan, and several European nations. The sample sizes ranged from 18 to 220. Most studies enrolled patients of both sexes, with three trials including only female patients. Early postoperative rehabilitation began within 3 days after surgery. The intervention frequency varied from 3 to 5 sessions daily to 3 sessions weekly, and the intervention duration ranged from 3 days to 6 months. The detailed study characteristics are provided in Table 1.

**Table 1:** Characteristics of included studies.

Study	Participants (T vs. C)				Interventions					Comparator	Outcomes	Results
	Country	Sample size	Age	Women/men	Type	Starting time	Duration	Frequency	Length			
Jin et al. (32)	China	33 vs. 33	66.45 ± 3.49 vs. 66.30 ± 4.41	18/15 vs. 17/16	VR-based rehab	POD 2	30 min/session	3 times/day	14 days	CPT	WOMAC, VAS	VR intervention aided rehab, reduced post-op pain, improved functional recovery.
Maeda et al. (33)	Japan	40 vs. 36	74.2 ± 6.3 vs. 74.0 ± 5.1	34/6 vs. 31/5	HAL-SJ + CPT	POD 1	NA	5 times/week	14 days	Active KE + CPT	VAS	Knee extension using HAL-SJ more effectively improved acute knee pain and extension angle.
Stasi et al. (34)	Greece	25 vs. 25 vs. 25	75.04 ± 4.61 vs. 71.72 ± 6.24 vs. 72.64 ± 6.15	16/9 vs. 18/7 vs. 22/3	CPT + CPM / CPM	POD 2	60 min or 30 min/session	2 times/day	6 days	CPT	ROM, VAS	CPM increased post-op knee flexion, but no significant difference in extension or pain.
Lee et al. (35)	Korea	19 vs. 19	72.05 ± 5.15 vs. 71.89 ± 5.44	19/0 vs. 19/0	Progressive dynamic balance + CPT	POD 3	30 min/session	5 times/week	42 days	CPT	WOMAC, ROM	Combined CPT and balance training positively affected physical function, balance, QoL.
Candiri et al. (36)	Turkey	9 vs. 9	64.77 ± 4.52 vs. 67.22 ± 6.61	8/1 vs. 8/1	GMI + CPT	POD1-2	10 min/session	3 times/week	42 days	CPT	VAS, WOMAC, ROM	GMI improved pain and muscle strength, but no significant functional improvement observed.
Schulz et al. (37)	Germany	25 vs. 25	69 ± 8 vs. 71 ± 8	12/13 vs. 14/11	CAM	POD 2	NA	NA	40 days	CPM	VAS, ROM	Both improved, CAM superior for flexion, pain, QoL.
Shim et al. (38)	Korea	27 vs. 27	68.25 ± 5.80 vs. 72.96 ± 4.56	23/5 vs. 21/7	AR-based home training + CPT	POD 0	30 min/session	1 session/day	84 days	CPT	WOMAC, NRS	Digital system improved function, pain, QoL, non-inferior to CPT, high satisfaction.
Hardt et al. (39)	Germany	33 vs. 27	66.3 ± 9.3 vs. 58.5 ± 10.3	18/15 vs. 16/11	App-based active training + CPT	POD 0	5 min/session	3–5 times/day	7 days	CPT	NRS	App-based training improved active ROM, reduced pain, enhanced strength early post-op.
Wirries et al. (40)	Germany	20 vs. 20	68.0 ± 9.2 vs. 67.4 ± 9.2	16/4 vs. 13/7	CPM + CPT	POD 1	60 min/session	2 times/day	NA	CPT	ROM, WOMAC	CPM improved early flexion and passive ROM, no long-term benefit at 2y.
Paravlic et al. (41)	Slovenia	13 vs. 13	61.69 ± 5.19 vs. 58.85 ± 5.24	6/7 vs. 6/7	MI + CPT	POD 2	15 min/session	5 times/week	28 days	CPT	ROM, VAS	MI group showed less strength loss, better TUG, no ROM difference at 1 month.
Jiao et al. (42)	China	39 vs. 39	75 ± 7.4 vs. 76 ± 8.2	32/7 vs. 35/4	High-intensity progressive rehab	Peri-op	NA	3–4 times/day	NA	CPT	VAS	Intervention group had lower VAS pain scores and higher satisfaction.
Jiao et al. (43)	China	39 vs. 39	74.6 ± 6.0 vs. 76.0 ± 6.3	26/13 vs. 30/9	ERAS-based quantitative rehab	Peri-op	NA	NA	NA	CPT	VAS	ERAS protocol safe, effective, accelerated joint functional recovery.
Briones-Cantero et al. (62)	Spain	12 vs. 12	73 ± 5 vs. 72 ± 6	4/8 vs. 5/7	MI + CPT	POD 1	30 min/session	1 time/day	5 days	CPT	VAS, WOMAC	MI group showed greater pain improvement, no ROM difference.
Nishitha et al. (44)	India	18 vs. 18	51.2 ± 5.2	21/15	VR-based rehab	POD 0	40 min/session	3 times/week	84 days	High-intensity exercise	NPRS, ROM, WOMAC	VR rehab more effective for pain, ROM, gait, functional independence.
Bid et al. (45)	India	17 vs. 17	50–75	13/4 vs. 15/2	CPT + CPM	POD 1	240 min/session	5 times/week	56 days	CPT	NPRS, ROM	CPM enhanced early knee mobility and function, no effect on pain and strength.
Sahni et al. (46)	India	29 vs. 25	69.5 ± 5.1 vs. 70.5 ± 5.1	20/9 vs. 18/7	CPM + CPT	POD 1	150 min/session	1 session/day	15 days	CPT	ROM, WOMAC	CPM showed no clinical improvement but positive effects on subjective pain, stiffness, function.
Baloch et al. (47)	Pakistan	38 vs. 38	61.6 ± 9.1 vs. 65.5 ± 7.9	32/6 vs. 29/9	CPT + CPM	POD 1	60 min/session	2 times/day	Approximately 6–9 days	CPT	ROM	CPM had no significant effect on knee ROM at discharge.
An et al. (48)	Korea	20 vs. 20	71.60 ± 3.53 vs. 70.04 ± 2.47	20/0 vs. 20/0	CCE	POD 3	30 min/session	5 times/week	28 days	OKCE	WOMAC,	CCE more effective in improving physical function, balance, dynamics.
Shabbir et al. (49)	Pakistan	31 vs. 33	60 ± 10 vs. 71 ± 12	NA	FT + CPT	POD 1	NA	5 times/week	28 days	RT	VAS	Functional training resulted in greater pain reduction.
Eymir et al. (50)	Turkey	58 vs. 55	68.9 ± 8.9 vs. 68.9 ± 8.3	49/9 vs. 50/5	AHSE + CPT	POD 1	30 min/session	2 times/day	Approximately 5–6 days	CPM + CPT	NPRS, ROM	AHSE group superior for pain, function, proprioception.
Yasaci et al. (51)	Turkey	22 vs. 22	64.22 ± 6.63 vs. 66.72 ± 5.62	16/4 vs. 19/1	Easy-Flex device + CPT	POD 1	T: 30–40 min Easy-Flex + 20 min CPT/session; C: 50–60 min/session	EFG: 2 times/day; CPT: 1 time/day	NA	CPT	ROM, NPRS, WOMAC	EF group superior for ROM, pain, function, QoL.
An et al. (52)	Korea	19 vs. 19	73.6 ± 4.8 vs. 72.3 ± 4.6	19/0 vs. 19/0	Comprehensive balance + CPT	POD 3	30 min/session	5 times/week	28 days	CPT	WOMAC, ROM	Balance training + CPT improved ROM, balance, gait, function more effectively.
Núñez-Cortés et al. (21)	Spain	20 vs. 20	70.6 ± 6.9 vs. 71.6 ± 7.2	9/11 vs. 8/12	High-intensity elastic resistance	POD 1	40 min/session	1 session/day	3 days	CPT	WOMAC, VAS, ROM	Three sessions improved physical function, pain, ROM significantly.
Goetz et al. (53)	Germany	30 vs. 22	66.97 ± 9.30 vs. 66.95 ± 8.44	16/14 vs. 12/10	ERAS-based quantitative rehab	POD 0	NA	T: 2 times/day vs. C: 1 time/day	NA	CPT	NRS	Significantly better peak torque, work, power at 5d; no difference at 4w. Both groups satisfied.
Yu et al. (54)	Korea	12 vs. 12	69.54 ± 3.12 vs. 68.39 ± 4.24	11/1 vs. 10/2	AR + CPT	POD 3	30 min/session	3 times/week	28 days	CPT + CPM	ROM, VAS	Significant improvements in both groups, no intergroup differences.
Arslan et al. (55)	Pakistan	13 vs. 13	65.00 ± 8.77 vs. 65.69 ± 7.9	NA	Structured rehab + CPT	POD 1	NA	Total 16 sessions	28 days	CPT	NPRS, WOMAC	Both improved, experimental group showed greater improvement in pain, function, ROM.
Cook et al. (56)	USA	19 vs. 19	64.4 ± 9.3 vs. 62.8 ± 7.2	9/10 vs. 12/7	Mizzou Biojoint Flex device+ CPT	POD 1	NA	NA	90 days	CPT	VAS	MBF group significantly better for ROM, pain, function, satisfaction at 3 months.
Bäcker et al. (57)	Germany	20 vs. 15	62.95 ± 8.25 vs. 66.27 ± 10.57	12/8 vs. 9/6	App-based (GenuSport) exercises	POD 0	5 min/session	3–5 times/day	42 days	CPT	VAS	App-based exercise showed significant improvement in VAS pain.
Joshi et al. (58)	USA	50 vs. 55	68.5 ± 7.8 vs. 70.5 ± 8.7	30/20 vs. 42/13	CPM + CPT	POD 1	120 min/session	3 times/day	NA	CPT	ROM, WOMAC	No significant differences in ROM or outcomes.
Kondo et al. (59)	Japan	34 vs. 35	75.8 ± 5.8 vs. 75.5 ± 6.1	28/6 vs. 29/6	Isometric quad exercise with auditory and visual feedback	POD 2	10-s contraction, 10-s rest (2 sets of 10 repetitions)	1 session/day (Days 2–3), 2 sessions/day (Days 4–14)	13 days	CPT	VAS, ROM, WOMAC	Intervention group had less pain at W1,2,3; better TUG, WOMAC, pain, function at W3.
Fitz et al. (60)	USA	38 vs. 45	61.5 ± 8.8 vs. 64.7 ± 9.2	20/18 vs. 31/14	CAM	POD 1	T: 20 min/session C: 240 min/session	3 times/day	21 days	CPM	WOMAC, ROM	CAM group showed better improvement across all metrics.
Gil-González et al. (61)	Spain	105 vs. 115	74.23 ± 6.79 vs. 73.33 ± 6.9	67/38 vs. 70/45	CPM + CPT	POD 0	60 min/session	3 times/day	NA	CPT	ROM, VAS	CPM provided no benefit for knee flexion or pain.

C, control group; T, treatment group; NA, not available; CPT, conventional physical therapy; HAL-SJ, Hybrid Assistive Limb single-joint; CPM, continuous passive motion; VR, virtual reality; GMI, graded motor imagery; CAM, controlled active movement; AR, augmented reality; MI, motor imagery; ERAS, enhanced recovery after surgery; CCE, combined kinematic chain exercise; OKCE, open kinetic chain exercise; AHSE, active heel slide exercise; ROM, range of motion; POD, postoperative day; VAS, Visual Analog Scale; WOMAC, Western Ontario and McMaster Universities Osteoarthritis Index; NPRS, Numeric Pain Rating Scale; NRS, Numeric Rating Scale; QoL, quality of life.

The exercise intervention measures were categorized as follows: conventional physical therapy (CPT), active range of motion (ROM) training, resistance training, functional training, CPM, device-assisted active training, intelligence-assisted training, ERAS-based rehabilitation pathway, and motor imagery. The classification of intervention nodes was based on commonly used clinical rehabilitation categories and the actual intervention contrasts reported in the included RCTs. In clinical practice and rehabilitation trials, early post-TKA exercise interventions are often distinguished not only by training content, such as functional or resistance training, but also by the mode of delivery, such as digital or device-assisted training, and by structured perioperative rehabilitation pathways, such as ERAS-based rehabilitation. Therefore, each study arm was assigned to a node according to the main therapeutic component that distinguished the experimental intervention from the comparator. For example, interventions primarily focusing on task-oriented movement, balance, gait, or functional activities were classified as functional training; those mainly emphasizing progressive muscle strengthening or external resistance were classified as resistance training; and interventions delivered through digital, virtual reality, augmented reality, app-based, or feedback-based systems were classified as intelligence-assisted training. When a rehabilitation program included multiple components, the classification was determined according to the dominant component and the principal contrast between the intervention and control groups. Two researchers independently performed node classification, and any disagreements were resolved through discussion. Detailed operational definitions and representative examples for each intervention node are provided in Supplementary Information S3.

### Assessment of the risk of bias

**Figure 2:**
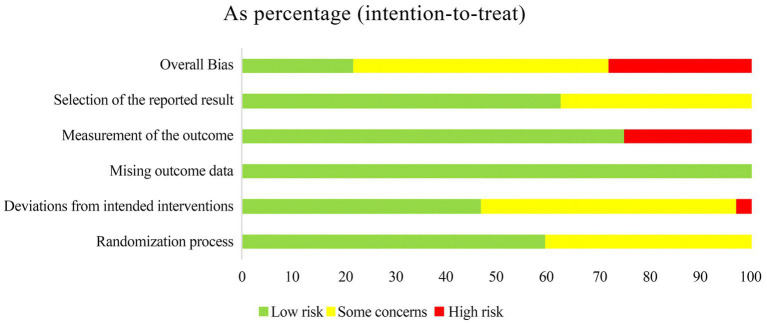
Schematic diagram of methodological bias risk assessment for studies included in this research.

**Figure 3:**
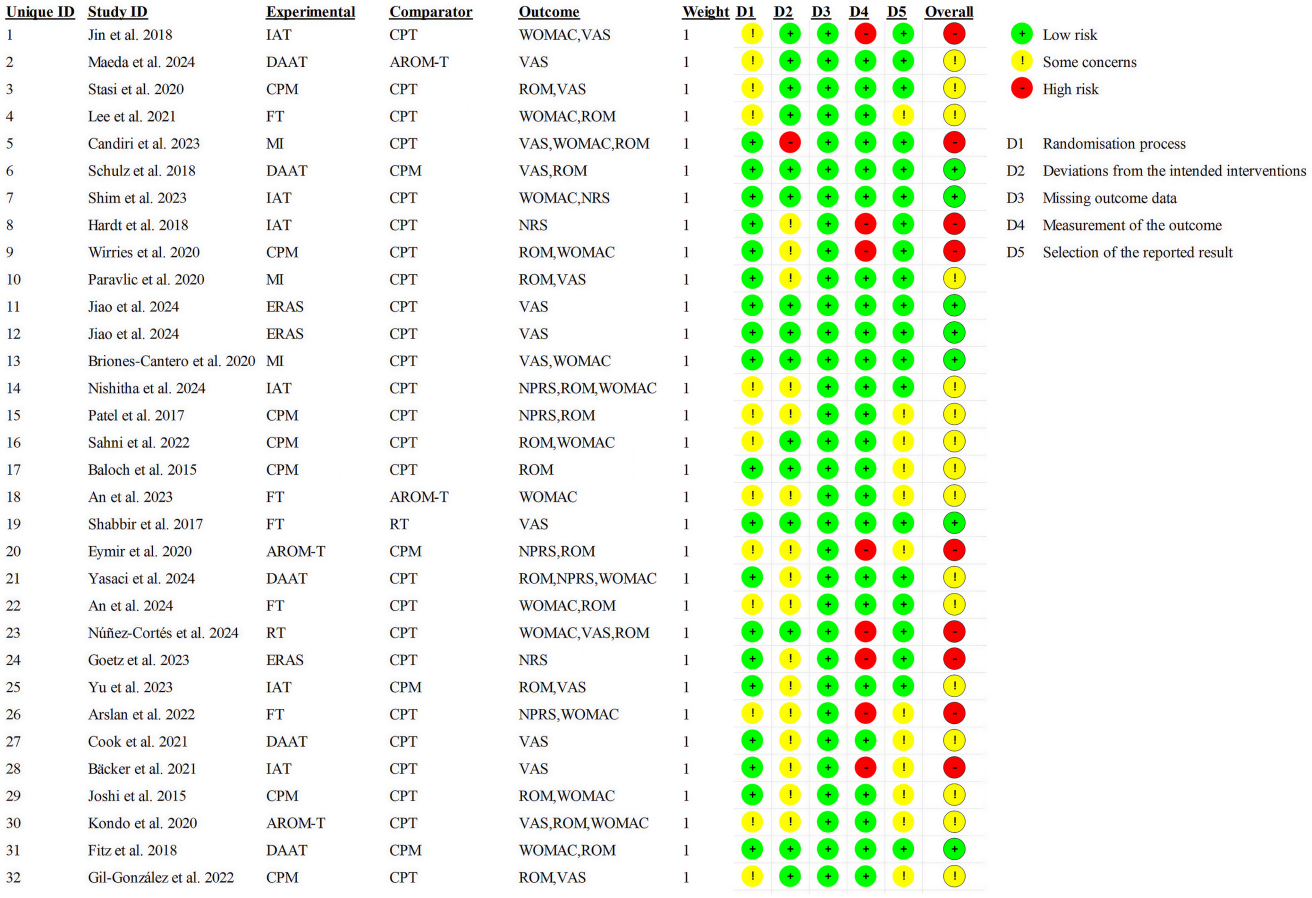
Proportion chart of each item in the bias risk assessment for this study.

The methodological quality of the 32 included studies was assessed using the Cochrane Risk of Bias tool (RoB2). On the basis of assessment using RoB2, 7 studies were judged to have a low overall risk of bias, 16 raised some concerns, and 9 were rated as having high risk. Although most studies performed adequately in terms of randomization and handling of missing data, risks were identified in domains such as allocation concealment, blinding, and selective reporting. A summary of these assessments is presented in Figures 2, 3, with the detailed results available in Supplementary Information S5.

### Pain outcomes

**Figure 4:**
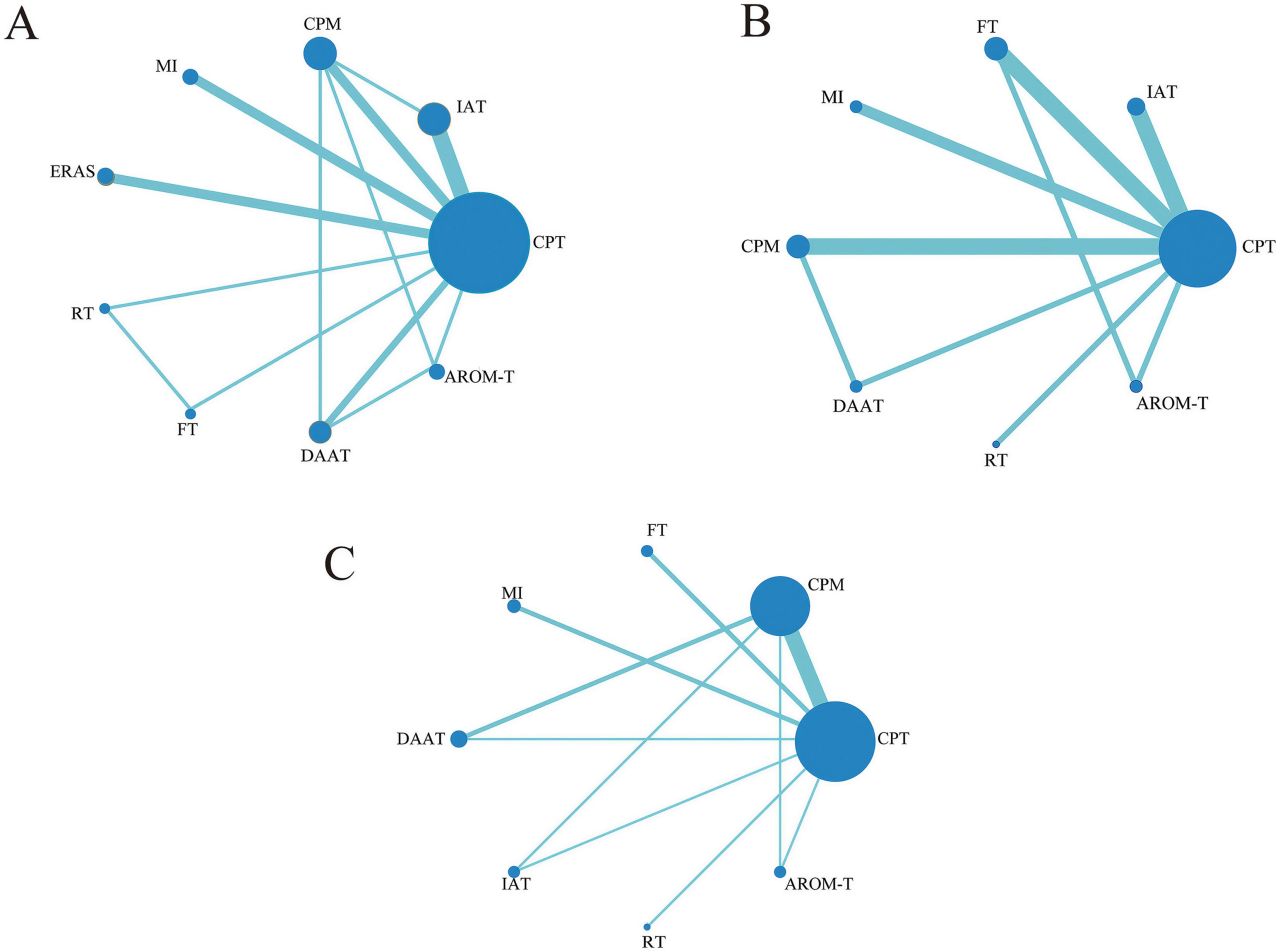
Network meta-analysis of eligible comparisons for **(A)** pain, **(B)** WOMAC, and **(C)** ROM. Each node represents an intervention, and the connecting line between two nodes indicates one or more RCTs directly comparing the two interventions. The size of each node is proportional to the number of randomly assigned participants, and the thickness of the line connecting two nodes is weighted according to the number of RCTs directly comparing the connected interventions.

Twenty-four studies reported pain outcomes (21, 32–34, 36–39, 41–45, 49–51, 53–57, 59, 61, 62). The comparative network (Figure 4A) includes nine nodes, with CPT as the central control in a closed evidence loop. Direct pairwise meta-analysis revealed that early exercise therapy significantly reduced pain compared with the control (SMD = −0.38, 95% CI: −0.50 to −0.25, *I*^2^ = 76.1%) (Supplementary Figure 2).

**Figure 5:**
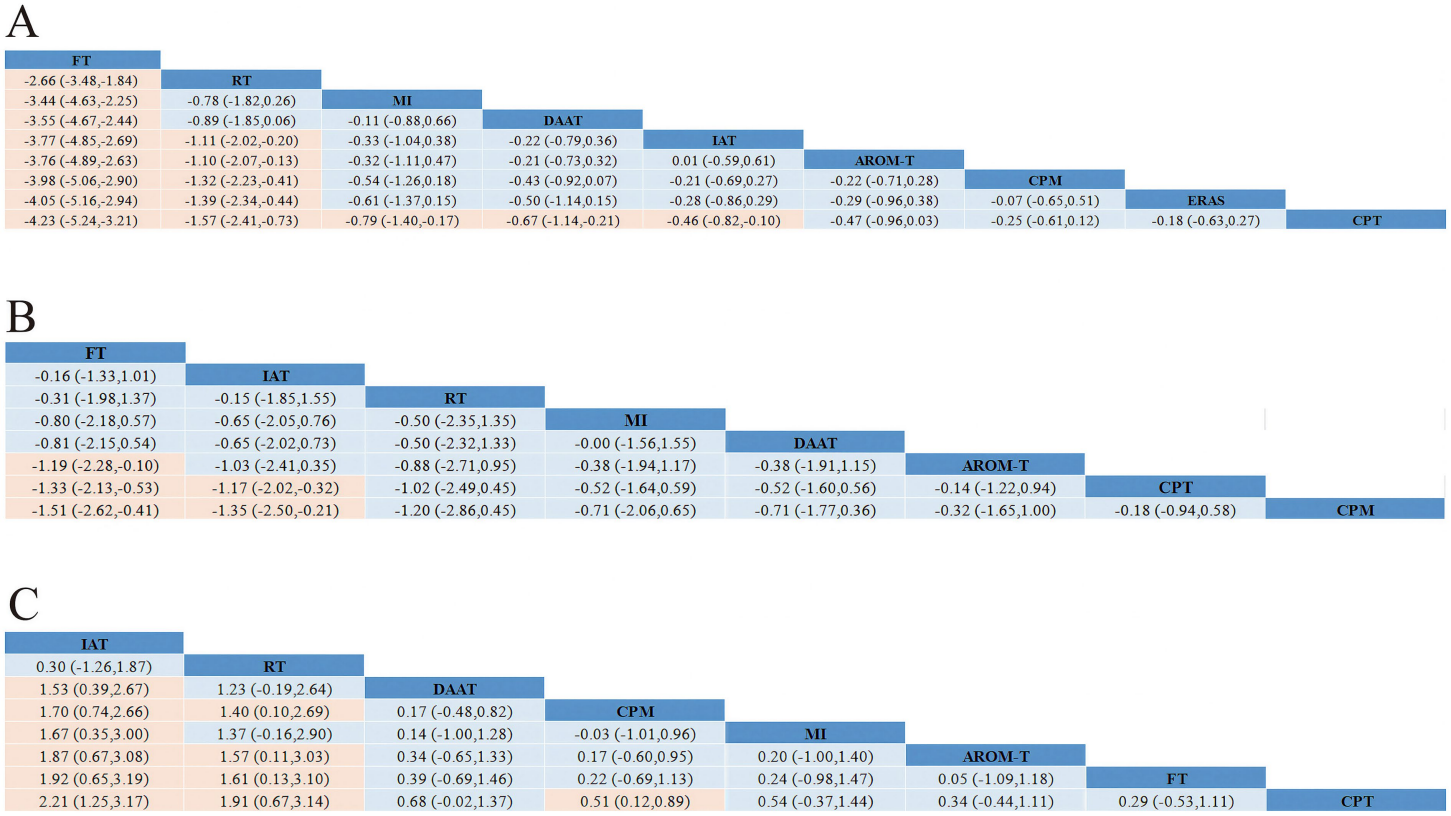
Comparison of effectiveness results for **(A)** pain, **(B)** WOMAC, and **(C)** ROM. Each cell displays the SMD with its 95% CI. For pain and WOMAC, a negative SMD indicates that the intervention in the upper-left corner is more effective, whereas for ROM, a positive SMD indicates that the intervention in the upper-left corner is more effective. Results with statistically significant differences are highlighted in red.

**Figure 6:**
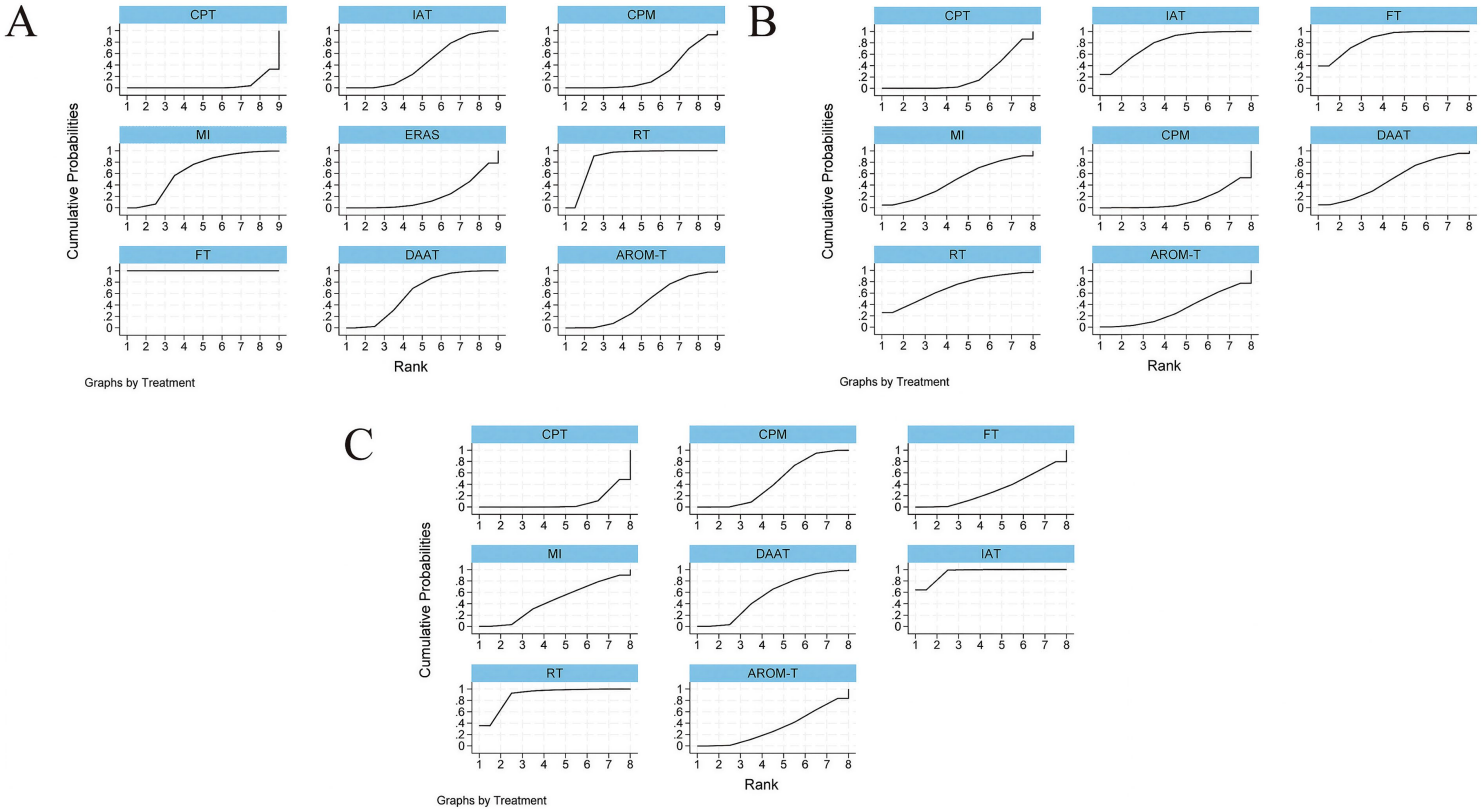
Cumulative ranking probability plots for **(A)** pain, **(B)** WOMAC, and **(C)** ROM. The horizontal axis represents the possible ranking of each treatment (from best to worst based on the outcome). The vertical axis represents the cumulative probability of each treatment being the best intervention, the second-best intervention, the third-best intervention, and so forth.

According to the random-effects model, functional training was associated with a larger reduction in pain compared with CPT (SMD = −4.23, 95% CI: −5.24 to −3.21), followed by resistance training (Figure 5A). Motor imagery, device-assisted active training, and intelligence-assisted training also showed statistically significant advantages over CPT, whereas active ROM training, CPM, and ERAS-based rehabilitation pathway showed no statistically significant advantage in pain reduction. The SUCRA rankings (Figure 6A; Table 2) suggested that functional training had the highest ranking probability (SUCRA = 100%), followed by resistance training (85.8%), motor imagery (64.7%), and device-assisted active training (60.5%), whereas CPT had the lowest ranking probability (4.6%). Global inconsistency was nonsignificant (χ^2^ = 1.54; *p* = 0.909), and node splitting revealed no local inconsistency (*p* > 0.05) (Supplementary Figure 1). The contour plots indicated that the evidence was derived mainly from direct comparisons such as CPT vs. CPM and CPT vs. resistance training (Supplementary Figure 3). Funnel plots revealed basic symmetry, suggesting no major publication bias (Supplementary Figure 4). For pain outcomes, the certainty of evidence was rated as moderate according to CINeMA. The certainty was downgraded mainly because of concerns related to risk of bias and heterogeneity, whereas no downgrading was applied for publication bias, indirectness, imprecision, or inconsistency (Supplementary Table 5).

**Table 2:** Cumulative rank probability table of different exercise types on pain, WOMAC, and ROM.

Exercise	Pain			WOMAC			ROM		
	SUCRA	PrBest	MeanRank	SUCRA	PrBest	MeanRank	SUCRA	PrBest	MeanRank
CPT	4.6	0.0	8.6	21.6	0.0	6.5	8.7	0.0	7.4
IAT	44.1	0.0	5.5	78.7	24.6	2.5	94.7	64.2	1.4
CPM	25.6	0.0	6.9	14.0	0.0	7.0	45.0	0.0	4.9
MI	64.7	0.0	3.8	49.1	4.8	4.6	44.9	0.2	4.9
ERAS	20.8	0.0	7.3	–	–	–	–	–	–
RT	85.8	0.0	2.1	68.5	25.9	3.2	88.9	35.5	1.8
FT	100.0	100.0	1.0	85.5	39.5	2.0	31.0	0.0	5.8
DAAT	60.5	0.0	4.2	51.1	4.9	4.4	54.4	0.1	4.2
AROM-T	43.9	0.0	5.5	31.5	0.4	5.8	32.3	0.0	5.7

A higher SUCRA value corresponds to a lower average ranking, indicating better treatment efficacy.

### WOMAC outcomes

Sixteen studies reported WOMAC scores (21, 32, 35, 36, 38, 40, 44, 46, 48, 51, 52, 55, 58–60, 62). The comparative network (Figure 4B) includes eight nodes, with CPT as the central control in a well-connected structure. Direct meta-analysis revealed that compared with the control, early exercise therapy significantly improved functional status based on WOMAC scores (SMD = −0.60, 95% CI: −1.03 to −0.18, *I*^2^ = 84.5%) (Supplementary Figure 6).

The results (Figure 5B) showed that, compared with CPT, functional training (SMD = −1.51, 95% CI: −2.62 to −0.41) and intelligence-assisted training (SMD = −1.35, 95% CI: −2.50 to −0.21) were associated with greater improvements in WOMAC scores, whereas CPM showed a comparatively smaller effect. Based on SUCRA values (Figure 6B; Table 2), functional training had the highest ranking probability (85.5%), followed by intelligence-assisted training (78.7%) and resistance training (68.5%), while CPM had the lowest ranking probability (14.0%). Global inconsistency was nonsignificant (χ^2^ = 1.94; *p* = 0.380), and node splitting indicated consistency across all local comparisons (Supplementary Figure 5). According to the contribution analysis, the direct comparison of CPT vs. functional training contributed the most to the network estimate (Supplementary Figure 7). The funnel plots showed no substantial asymmetry, suggesting minimal publication bias (Supplementary Figure 8). For WOMAC outcomes, the certainty of evidence was rated as low. The downgrading was mainly attributable to concerns regarding risk of bias, indirectness, and heterogeneity. No downgrading was applied for publication bias, imprecision, or inconsistency (Supplementary Table 5).

### ROM outcomes

Nineteen studies reported ROM outcomes (21, 34–37, 40, 41, 44–47, 50–52, 54, 58–61). The network diagram (Figure 4C) comprises eight nodes, with the most direct comparisons observed between CPT and CPM. Direct pairwise meta-analysis indicated that compared with the control treatment, early exercise therapy significantly improved ROM (SMD = 0.69, 95% CI 0.33–1.05, *I*^2^ = 83.4%) (Supplementary Figure 10).

The results (Figure 5C) indicated that, compared with CPT, intelligence-assisted training (SMD = 2.21, 95% CI: 1.25–3.17) and resistance training (SMD = 1.91, 95% CI: 0.67–3.14) showed more favorable effects on postoperative ROM improvement. CPM also demonstrated a significant benefit over CPT (SMD = 0.51, 95% CI: 0.12–0.89). According to the SUCRA-based ranking probabilities (Figure 6C; Table 2), intelligence-assisted training had the highest probability of being ranked favorably (94.7%), followed by resistance training (88.9%) and device-assisted active training (54.4%), whereas CPT had the lowest ranking probability (8.7%). No significant global inconsistency was observed (χ^2^ = 1.78; *p* = 0.620), and node splitting revealed no local inconsistencies (Supplementary Figure 9). According to the contribution analysis, direct comparisons between CPT and functional training and between CPT and motor imagery contributed the most to the network estimate (Supplementary Figure 11). Funnel plots showed near symmetry, suggesting minimal publication bias (Supplementary Figure 12). For ROM outcomes, the certainty of evidence was rated as low. Similar to WOMAC outcomes, the certainty was downgraded mainly because of concerns related to risk of bias, indirectness, and heterogeneity, while publication bias, imprecision, and inconsistency did not lead to downgrading (Supplementary Table 5).

## Discussion

This study employed an NMA approach to systematically compare the efficacy of various early exercise interventions initiated within the “golden period” of 3 days post-TKA for patient pain, WOMAC scores, and ROM. It provided high-level evidence for ranking these interventions. In addition, the results offer evidence-based guidance for the precise implementation of postoperative rehabilitation.

### Pain

The results showed that functional training and resistance training were associated with more favorable effects on pain relief than the control intervention. Functional training comprises strength-based training, balance drills, and task-oriented practice. Biomechanically, functional training emphasizes coordinated, multijoint movements, which may help correct faulty movement patterns and reduce the risks of secondary pain and joint degeneration caused by abnormal loading, as suggested by previous research (63). Furthermore, functional training shifts the goals of rehabilitation from passive management to active task completion, which may in turn enhance patient self-efficacy—a key factor linked to reducing the intensity of chronic pain and improving patient outcomes (64). Conversely, resistance training may activate widespread “exercise-induced hypoalgesia,” potentially promoting endogenous opioid release and activating descending inhibitory pathways to systemically increase pain thresholds (65). Postoperatively, functional training may also influence the balance of inflammatory cytokines by inhibiting the synthesis of TNF-*α* and promoting the production of anti-inflammatory cytokines such as IL-10; maintaining this balance might contribute to reducing systemic inflammation, further relieving pain symptoms (66).

The greater analgesic effect of functional training over isolated strength training likely stems from its inherently multimodal nature. Unlike strength training, which focuses primarily on isolated muscle strengthening, functional training programs typically incorporate task-specific movements, proprioceptive exercises, and neuromuscular control drills alongside conventional strength exercises, thereby engaging both peripheral and central pain modulatory mechanisms (67, 68). Whereas strength training mainly enhances quadriceps strength and joint stability, functional training additionally improves movement quality, gait mechanics, and motor coordination, collectively reducing abnormal joint loading and nociceptive sensitization (69). Furthermore, by embedding strength exercises within functional movement patterns, such multimodal rehabilitation may foster greater patient engagement and adherence, thereby further amplifying pain relief outcomes (70).

### Joint function

The results showed that functional training and intelligence-assisted training appeared to provide greater improvements in WOMAC-based functional status than the control intervention. Functional training showed a relatively large short-term effect estimate (SMD = −1.51) and a high SUCRA-based ranking probability (85.5%) among the included interventions. Following TKA, patients often develop abnormal movement patterns due to pain, swelling, and weakness. By simulating daily activities and integrating comprehensive training, functional training may optimize neuromuscular control (71). Through task-oriented repetition, functional training may activate knee-stabilizing muscles, promote sensorimotor integration, and enhance central regulation, which may in turn reduce the knee load (35). It may also improve balance and gait, which are crucial to fall prevention (8). Furthermore, functional training may promote the release of brain-derived neurotrophic factor (BDNF), thereby enhancing plasticity in motor-related brain regions (72, 73).

Intelligence-assisted training also showed a favorable ranking probability (SUCRA = 78.7%), which was attributed to cognitive and neurophysiological mechanisms. First, it provides real-time visual feedback that may help immediately correct joint angles (74, 75). Second, its immersive nature may enhance patient motivation and compliance—key factors for recovery (76). Third, the interactive tasks integrated into this training may activate higher-order functions such as spatial perception, thereby stimulating prefrontal networks and promoting neuroplasticity (77–79). Finally, intelligence-assisted training facilitates personalized rehabilitation given that parameters are adjusted to meet individual needs (80).

In contrast, CPM exhibited the least significant effect (SUCRA = 14.0%). As a passive modality, CPM may help maintain early ROM but may elicit only minimal muscle activation (81). While it may aid in healing, it may not fully replace active, task-oriented neuromuscular training (82, 83).

### Rom

For ROM recovery, intelligence-assisted training and resistance training showed more favorable comparative effects, with intelligence-assisted training having the highest SUCRA-based ranking probability (94.7%). The advantages of intelligence-assisted training are multifaceted; it may shift attention away from pain toward task performance, potentially reducing pain perception and helping enable active range attainment (84). Unlike passive stretching, intelligence-assisted training requires active patient-driven movement, which may promote synergistic muscle contraction and joint stability (85). With real-time feedback, patients can control movement amplitude, which may help prevent movement limitations due to fear (86). Moreover, resistance training may primarily enhance the mechanical basis for movement by increasing muscle strength and tendon stiffness, thereby supporting active mobility (87, 88).

An important methodological assumption of NMA is transitivity, which requires that the included trials are sufficiently comparable with respect to potential effect modifiers. In the present study, the transitivity assumption was considered clinically reasonable because all included studies enrolled patients undergoing TKA, evaluated early postoperative exercise-based or exercise-oriented rehabilitation initiated within 3 days after surgery, and reported common recovery outcomes, including pain, WOMAC scores, and ROM. In addition, intervention nodes were assigned according to the dominant therapeutic component and the principal contrast between trial arms, which helped maintain clinical interpretability across comparisons. The statistical assessment also supported the overall coherence of the network, as no significant global or local inconsistency was detected across the main outcomes. Moreover, the main findings were generally consistent with clinical rehabilitation principles: functional training showed relatively consistent advantages for pain relief and WOMAC-based functional improvement, whereas intelligence-assisted training and resistance training showed greater potential for ROM improvement. Nevertheless, variations in intervention frequency, duration, supervision, rehabilitation intensity, perioperative care, and co-interventions across studies may still represent potential effect modifiers. Therefore, the comparative effects and SUCRA rankings should be interpreted as evidence for the relative performance of early rehabilitation strategies under the current evidence network, rather than as definitive superiority under all clinical settings.

This study has several important strengths. First, it systematically compared and ranked nine early exercise interventions initiated within the 3-day postoperative period after TKA, thereby addressing an important evidence gap. Second, the inclusion of 32 randomized controlled trials comprising 1,871 patients, together with a clearly defined classification of intervention nodes, supported the construction of a connected evidence network. Third, the CINeMA framework was used to assess the certainty of evidence, and the review was conducted according to PRISMA-NMA guidelines, thereby improving methodological transparency.

From a clinical perspective, the present findings may provide useful comparative information for physiotherapists when selecting early rehabilitation strategies after TKA. Intelligence-assisted training showed statistically significant comparative benefits over CPT across all three outcomes, whereas functional training showed favorable comparative effects for both pain relief and WOMAC-based functional improvement. Resistance training also appeared promising, particularly for pain relief and ROM recovery. However, these findings should be interpreted in combination with effect estimates, confidence intervals, heterogeneity, and CINeMA certainty ratings, rather than on the basis of SUCRA rankings alone. In the CINeMA assessment, downgrading was mainly due to risk-of-bias concerns and heterogeneity for pain outcomes, and to risk of bias, indirectness, and heterogeneity for WOMAC and ROM outcomes. Therefore, the results should be regarded as supportive evidence for individualized clinical decision-making rather than as definitive proof that one intervention is optimal across all clinical contexts.

## Limitations

This study has several limitations. First, variability in intervention protocols (intensity, frequency, duration) across RCTs may affect precision. Second, differences in outcome measures limit comparability. Third, the blinding of participants and therapists was often inadequate, leading to potential bias. Additionally, the included studies did not report the specific protocols or implementation of preoperative prehabilitation, precluding the assessment of the influence of this confounding factor on the results. Finally, most studies did not adequately control for confounders such as therapist expertise or psychological factors. Large-sample RCTs that directly compare modalities and standardize reporting should be conducted.

## Conclusion

This NMA compared early exercise-based rehabilitation interventions initiated within 3 days after TKA. For postoperative pain relief, functional training and resistance training showed favorable comparative effects, with moderate certainty of evidence. For WOMAC-based functional status, functional training and intelligence-assisted training appeared to be associated with greater improvement, although the certainty of evidence was low. For ROM recovery, intelligence-assisted training and resistance training showed favorable comparative effects, with low certainty of evidence.

Overall, intelligence-assisted training showed comparative benefits across pain, function, and ROM, while functional training showed relatively consistent advantages for pain relief and functional improvement. These findings may help inform individualized early rehabilitation decisions according to patients’ clinical conditions, rehabilitation goals, and available resources. Further high-quality RCTs with standardized intervention protocols are needed to confirm these comparative findings.

## Data Availability

The datasets analyzed during the current study are available from the corresponding author on reasonable request.

## Glossary

TKA: Total Knee Arthroplasty
KOA: Knee Osteoarthritis
ROM: Range of Motion
WOMAC: Western Ontario and McMaster Universities Osteoarthritis Index
ERAS: Enhanced Recovery After Surgery
LOS: Length of Stay
RCT: Randomized Controlled Trial
NMA: Network Meta-Analysis
SMD: Standardized Mean Difference
CI: Confidence Interval
SUCRA: Surface Under the Cumulative Ranking Curve
CPT: Conventional Physical Therapy
FT: Functional Training
RT: Resistance Training
IAT: Intelligence-Assisted Training
MI: Motor Imagery
CPM: Continuous Passive Motion
DAAT: Device-Assisted Active Training
AROM-T: Active Range of Motion Training
VAS: Visual Analog Scale
NRS: Numeric Rating Scale
NPRS: Numeric Pain Rating Scale
VR: Virtual Reality
AR: Augmented Reality
QoL: Quality of Life
POD: Postoperative Day
