# Supplementary material for: Comparative efficacy of early exercise interventions for recovery outcomes after total knee arthroplasty: a systematic review and network meta-analysis
Source: Front Med (Lausanne). 2026 Jul 17;13:1883684. doi: 10.3389/fmed.2026.1883684 (PMC13424120; doi:10.3389/fmed.2026.1883684)
Supplement: Supplementary file 1 [file Supplementary_file_1.docx]

**Supplementary Information**

[Supplementary Information S1. PRISMA checklist of current network meta-analysis 2](#_Toc421)

[Table 1 PRISMA checklist of current network meta-analysis 2](#_Toc3386)

[Supplementary Information S2. Search strategy 7](#_Toc2669)

[Table 2 Detailed search strategy in each database and search result 7](#_Toc14067)

[Supplementary Information S3. Included types of exercise, definitions, and core content 17](#_Toc31026)

[Table 3 Included types of exercise, definitions, and core content 17](#_Toc32366)

[Supplementary Information S4. The list for the included studies 20](#_Toc24703)

[Supplementary Information S5. Risk of bias of included studies 24](#_Toc9202)

[Table 4 Quality evaluation of the included literature 25](#_Toc8147)

[Supplementary Information S6. The analysis for primary outcomes of pain 29](#_Toc4079)

[Figure 1 Loop-specific heterogeneity for pain 31](#_Toc12557)

[Figure 2 Forest plot of pain outcome indicators 32](#_Toc25422)

[Figure 3 Contribution plot for pain 33](#_Toc3565)

[Figure 4 Funnel plot of pain outcome measures 34](#_Toc865)

[Supplementary Information S7. The analysis for primary outcomes of WOMAC 34](#_Toc22208)

[Figure 5 Loop-specific heterogeneity for WOMAC 36](#_Toc18013)

[Figure 6 Forest Plot of WOMAC Outcome Indicators 37](#_Toc27527)

[Figure 7 Contribution plot for WOMAC 38](#_Toc19577)

[Figure 8 Funnel plot of WOMAC outcome measures 39](#_Toc29446)

[Supplementary Information S8. The analysis for primary outcomes of ROM 39](#_Toc13094)

[Figure 9 Loop-specific heterogeneity for ROM 41](#_Toc26531)

[Figure 10 Forest Plot of ROM Outcome Indicators 42](#_Toc31492)

[Figure 11 Contribution plot for ROM 43](#_Toc16390)

[Figure 12 Funnel plot of ROM outcome measures 44](#_Toc32381)

[Supplementary Information S9. Assessment of evidence certainty 44](#_Toc14798)

[Table 5 CINeMA Assessment of evidence certainty 44](#_Toc1579)

# Supplementary Information S1. PRISMA checklist of current network meta-analysis

## Table 1 PRISMA checklist of current network meta-analysis

| **Section/Topic** | **Item #** | **Checklist Item** | **Reported on Page #** |
| --- | --- | --- | --- |
| **TITLE** |  |  |  |
| Title | 1 | Identify the report as a systematic review *incorporating a network meta-analysis (or related form of meta-analysis).* | Title |
|  |  |  |  |
| **ABSTRACT** |  |  |  |
| Structured summary | 2 | Provide a structured summary including, as applicable:  **Background:** main objectives  **Methods:** data sources; study eligibility criteria, participants, and interventions; study appraisal; and *synthesis methods, such as network meta-analysis.*  **Results:** number of studies and participants identified; summary estimates with corresponding confidence/credible intervals; *treatment rankings may also be discussed. Authors may choose to summarize pairwise comparisons against a chosen treatment included in their analyses for brevity.*  **Discussion/Conclusions:** limitations; conclusions and implications of findings.  **Other:** primary source of funding; systematic review registration number with registry name. | Abstract section |
|  |  |  |  |
| **INTRODUCTION** |  |  |  |
| Rationale | 3 | Describe the rationale for the review in the context of what is already known*, including mention of why a network meta-analysis has been conducted.* | Introduction, paragraphs 1~3 |
| Objectives | 4 | Provide an explicit statement of questions being addressed, with reference to participants, interventions, comparisons, outcomes, and study design (PICOS). | Abstract (Objectives); Introduction, paragraphs 4； |
|  |  |  |  |
| **METHODS** |  |  |  |
| Protocol and registration | 5 | Indicate whether a review protocol exists and if and where it can be accessed (e.g., Web address); and, if available, provide registration information, including registration number. | Materials and Methods,paragraphs 1 |
| Eligibility criteria | 6 | Specify study characteristics (e.g., PICOS, length of follow-up) and report characteristics (e.g., years considered, language, publication status) used as criteria for eligibility, giving rationale. *Clearly describe eligible treatments included in the treatment network, and note whether any have been clustered or merged into the same node (with justification).* | Materials and Methods, Inclusion and Exclusion Criteria |
| Information sources | 7 | Describe all information sources (e.g., databases with dates of coverage, contact with study authors to identify additional studies) in the search and date last searched. | Search strategy |
| Search | 8 | Present full electronic search strategy for at least one database, including any limits used, such that it could be repeated. | Search strategy and Supplementary Information S2 |
| Study selection | 9 | State the process for selecting studies (i.e., screening, eligibility, included in systematic review, and, if applicable, included in the meta-analysis). | Selection criteria and Result |
| Data collection process | 10 | Describe method of data extraction from reports (e.g., piloted forms, independently, in duplicate) and any processes for obtaining and confirming data from investigators. | Study Selection and Data Extraction |
| Data items | 11 | List and define all variables for which data were sought (e.g., PICOS, funding sources) and any assumptions and simplifications made. | Data extraction |
| **Geometry of the network** | **S1** | Describe methods used to explore the geometry of the treatment network under study and potential biases related to it. This should include how the evidence base has been graphically summarized for presentation, and what characteristics were compiled and used to describe the evidence base to readers. | Statistical methods |
| Risk of bias within individual studies | 12 | Describe methods used for assessing risk of bias of individual studies (including specification of whether this was done at the study or outcome level), and how this information is to be used in any data synthesis. | Quality assessment |
| Summary measures | 13 | State the principal summary measures (e.g., risk ratio, difference in means). *Also describe the use of additional summary measures assessed, such as treatment rankings and surface under the cumulative ranking curve (SUCRA) values, as well as modified approaches used to present summary findings from meta-analyses.* | Statistical methods |
| Planned methods of analysis | 14 | Describe the methods of handling data and combining results of studies for each network meta-analysis. This should include, but not be limited to:   - *Handling of multi-arm trials;* - *Selection of variance structure;* - *Selection of prior distributions in Bayesian analyses; and* - *Assessment of model fit.* | Statistical methods |
| **Assessment of Inconsistency** | **S2** | Describe the statistical methods used to evaluate the agreement of direct and indirect evidence in the treatment network(s) studied. Describe efforts taken to address its presence when found. | Statistical methods |
| Risk of bias across studies | 15 | Specify any assessment of risk of bias that may affect the cumulative evidence (e.g., publication bias, selective reporting within studies). | Statistical methods |
| Additional analyses | 16 | Describe methods of additional analyses if done, indicating which were pre-specified. This may include, but not be limited to, the following:   - Sensitivity or subgroup analyses; - Meta-regression analyses; - *Alternative formulations of the treatment network; and* - *Use of alternative prior distributions for Bayesian analyses (if applicable).* | Statistical methods |
|  |  |  |  |
| **RESULTS†** |  |  |  |
| Study selection | 17 | Give numbers of studies screened, assessed for eligibility, and included in the review, with reasons for exclusions at each stage, ideally with a flow diagram. | Results, Study Selection and Figure 1 |
| **Presentation of network structure** | **S3** | Provide a network graph of the included studies to enable visualization of the geometry of the treatment network. | Figure 4 |
| **Summary of network geometry** | **S4** | Provide a brief overview of characteristics of the treatment network. This may include commentary on the abundance of trials and randomized patients for the different interventions and pairwise comparisons in the network, gaps of evidence in the treatment network, and potential biases reflected by the network structure. | Figure 4 |
| Study characteristics | 18 | For each study, present characteristics for which data were extracted (e.g., study size, PICOS, follow-up period) and provide the citations. | Table 1 |
| Risk of bias within studies | 19 | Present data on risk of bias of each study and, if available, any outcome level assessment. | Results of Risk of Bias and Figure 3 and 4 and Supplementary Information S5 |
| Results of individual studies | 20 | For all outcomes considered (benefits or harms), present, for each study: 1) simple summary data for each intervention group, and 2) effect estimates and confidence intervals. *Modified approaches may be needed to deal with information from larger networks.* | Table 1 |
| Synthesis of results | 21 | Present results of each meta-analysis done, including confidence/credible intervals. *In larger networks, authors may focus on comparisons versus a particular comparator (e.g. placebo or standard care), with full findings presented in an appendix. League tables and forest plots may be considered to summarize pairwise comparisons.* If additional summary measures were explored (such as treatment rankings), these should also be presented. | Table 2, Figure 4~6, Supplementary Information S6-S8 |
| **Exploration for inconsistency** | **S5** | Describe results from investigations of inconsistency. This may include such information as measures of model fit to compare consistency and inconsistency models, *P* values from statistical tests, or summary of inconsistency estimates from different parts of the treatment network. | Results of inconsistency and Supplementary Information S6-S8 |
| Risk of bias across studies | 22 | Present results of any assessment of risk of bias across studies for the evidence base being studied. | Assessment of the Risk of Bias |
| Results of additional analyses | 23 | Give results of additional analyses, if done (e.g., sensitivity or subgroup analyses, meta-regression analyses*, alternative network geometries studied, alternative choice of prior distributions for Bayesian analyses,* and so forth). | Supplementary Information S6-S8 |
|  |  |  |  |
| **DISCUSSION** |  |  |  |
| Summary of evidence | 24 | Summarize the main findings, including the strength of evidence for each main outcome; consider their relevance to key groups (e.g., healthcare providers, users, and policy-makers). | Discussion, paragraphs 1 |
| Limitations | 25 | Discuss limitations at study and outcome level (e.g., risk of bias), and at review level (e.g., incomplete retrieval of identified research, reporting bias). *Comment on the validity of the assumptions, such as transitivity and consistency. Comment on any concerns regarding network geometry (e.g., avoidance of certain comparisons).* | Discussion, limitations |
| Conclusions | 26 | Provide a general interpretation of the results in the context of other evidence, and implications for future research. | Conclusions section |
|  |  |  |  |
| **FUNDING** |  |  |  |
| Funding | 27 | Describe sources of funding for the systematic review and other support (e.g., supply of data); role of funders for the systematic review. This should also include information regarding whether funding has been received from manufacturers of treatments in the network and/or whether some of the authors are content experts with professional conflicts of interest that could affect use of treatments in the network. | Funding section |

# Supplementary Information S2. Search strategy

## Table 2 Detailed search strategy in each database and search result

| Database | Search strategy | Results |
| --- | --- | --- |
| PubMed： | (((((((((((((((((((((((((((((((((((((((((((((((((((((((((("Exercise"[Mesh]) OR (rehabilitation[Title/Abstract])) OR (physiotherapy[Title/Abstract])) OR (Activities of daily Living[Title/Abstract])) OR (Early Ambulation[Title/Abstract])) OR (Exercise Therapy[Title/Abstract])) OR (Blood Flow Restriction Therapy[Title/Abstract])) OR (Training[Title/Abstract])) OR (Motion Therapy, Continuous Passive[Title/Abstract])) OR (Muscle Stretching Exercises[Title/Abstract])) OR (Telerehabilitation[Title/Abstract])) OR (Physical Therapy Modalities[Title/Abstract])) OR (Electric Stimulation Therapy[Title/Abstract])) OR (Hydrotherapy[Title/Abstract])) OR (Mirror Movement Therapy[Title/Abstract])) OR (Musculoskeletal Manipulations[Title/Abstract])) OR (Myofunctional Therapy[Title/Abstract])) OR (Exercises[Title/Abstract])) OR (Physical Exercise[Title/Abstract])) OR (Physical Exercises[Title/Abstract])) OR (Exercise, Aerobic[Title/Abstract])) OR (Aerobic Exercise[Title/Abstract])) OR (Aerobic Exercises[Title/Abstract])) OR (Exercises, Aerobic[Title/Abstract])) OR (Exercise, Isometric[Title/Abstract])) OR (Exercises, Isometric[Title/Abstract])) OR (Isometric Exercises[Title/Abstract])) OR (Isometric Exercise[Title/Abstract])) OR (Acute Exercise[Title/Abstract])) OR (Acute Exercises[Title/Abstract])) OR (Exercise, Acute[Title/Abstract])) OR (Exercises, Acute[Title/Abstract])) OR (Training, Exercise[Title/Abstract])) OR (Trainings, Exercise[Title/Abstract])) OR (Physical Activity[Title/Abstract])) OR (Activities, Physical[Title/Abstract])) OR (Activity, Physical[Title/Abstract])) OR (Physical Activities[Title/Abstract])) OR (Movement[Title/Abstract])) OR (Motion[Title/Abstract])) OR (Motor Activity[Title/Abstract])) OR (Cool-Down Exercise[Title/Abstract])) OR (Exergaming[Title/Abstract])) OR (Gymnastics[Title/Abstract])) OR (Physical Conditioning[Title/Abstract])) OR (Endurance Training[Title/Abstract])) OR (High-Intensity Interval Training[Title/Abstract])) OR (Periodization[Title/Abstract])) OR (Plyometric Exercise[Title/Abstract])) OR (Resistance Training[Title/Abstract])) OR (Running[Title/Abstract])) OR (Jogging[Title/Abstract])) OR (Swimming[Title/Abstract])) OR (Walking[Title/Abstract])) OR (Stair Climbing[Title/Abstract])) OR (Yoga[Title/Abstract])) OR (Tai Chi[Title/Abstract])) AND ((((((((((((((((((((((((((((((((("Arthroplasty, Replacement, Knee"[Mesh]) OR (Arthroplasties, Replacement, Knee[Title/Abstract])) OR (Arthroplasty, Knee Replacement[Title/Abstract])) OR (Knee Replacement Arthroplasties[Title/Abstract])) OR (Knee Replacement Arthroplasty[Title/Abstract])) OR (Replacement Arthroplasties, Knee[Title/Abstract])) OR (Replacement Arthroplasty, Knee[Title/Abstract])) OR (Replacement, Total Knee[Title/Abstract])) OR (Total Knee Replacement[Title/Abstract])) OR (Knee Replacement, Total[Title/Abstract])) OR (Knee Arthroplasty[Title/Abstract])) OR (Arthroplasty, Knee[Title/Abstract])) OR (Arthroplasties, Knee Replacement[Title/Abstract])) OR (Knee Arthroplasty, Total[Title/Abstract])) OR (Arthroplasty, Total Knee[Title/Abstract])) OR (Total Knee Arthroplasty[Title/Abstract])) OR (Arthroplasty, Replacement, Partial Knee[Title/Abstract])) OR (Unicompartmental Knee Arthroplasty[Title/Abstract])) OR (Arthroplasty, Unicompartmental Knee[Title/Abstract])) OR (Knee Arthroplasty, Unicompartmental[Title/Abstract])) OR (Unicompartmental Knee Replacement[Title/Abstract])) OR (Knee Replacement, Unicompartmental[Title/Abstract])) OR (Partial Knee Replacement[Title/Abstract])) OR (Knee Replacement, Partial[Title/Abstract])) OR (Unicondylar Knee Replacement[Title/Abstract])) OR (Knee Replacement, Unicondylar[Title/Abstract])) OR (Unicondylar Knee Arthroplasty[Title/Abstract])) OR (Arthroplasty, Unicondylar Knee[Title/Abstract])) OR (Knee Arthroplasty, Unicondylar[Title/Abstract])) OR (Partial Knee Arthroplasty[Title/Abstract])) OR (Arthroplasty, Partial Knee[Title/Abstract])) OR (Knee Arthroplasty, Partial[Title/Abstract])))) AND (((((Randomized controlled trials[Title/Abstract]) OR (Randomized[Title/Abstract])) OR (placebo[Title/Abstract])))) Filters: in the last 10 years | 1393 |
| Embase | No. Query Results Results Date  #9. #8 AND (2015:py OR 2016:py OR 2017:py OR 2018:py 2,237 25 Oct 2025  OR 2019:py OR 2020:py OR 2021:py OR 2022:py OR  2023:py OR 2024:py OR 2025:py)  #8. #3 AND #6 AND #7 3,037 25 Oct 2025  #7. 'randomized controlled trials':ab,ti OR 1,522,407 25 Oct 2025  'randomized':ab,ti OR 'placebo':ab,ti  #6. #4 OR #5 2,677,769 25 Oct 2025  #5. 'rehabilitation':ab,ti OR 'physiotherapy':ab,ti 2,421,144 25 Oct 2025  OR 'activities of daily living':ab,ti OR 'early  ambulation':ab,ti OR 'exercise therapy':ab,ti OR  'blood flow restriction therapy':ab,ti OR  'training':ab,ti OR 'motion therapy, continuous  passive':ab,ti OR 'muscle stretching  exercises':ab,ti OR 'telerehabilitation':ab,ti OR  'physical therapy modalities':ab,ti OR 'electric  stimulation therapy':ab,ti OR  'hydrotherapy':ab,ti OR 'mirror movement  therapy':ab,ti OR 'musculoskeletal  manipulations':ab,ti OR 'myofunctional  therapy':ab,ti OR 'exercises':ab,ti OR 'physical  exercise':ab,ti OR 'physical exercises':ab,ti OR  'exercise, aerobic':ab,ti OR 'aerobic  exercise':ab,ti OR 'aerobic exercises':ab,ti OR  'exercises, aerobic':ab,ti OR 'exercise,  isometric':ab,ti OR 'exercises, isometric':ab,ti  OR 'isometric exercises':ab,ti OR 'isometric  exercise':ab,ti OR 'acute exercise':ab,ti OR  'acute exercises':ab,ti OR 'exercise,  acute':ab,ti OR 'exercises, acute':ab,ti OR  'training, exercise':ab,ti OR 'trainings,  exercise':ab,ti OR 'physical activity':ab,ti OR  'activities, physical':ab,ti OR 'activity,  physical':ab,ti OR 'physical activities':ab,ti OR  'movement':ab,ti OR 'motion':ab,ti OR 'motor  activity':ab,ti OR 'cool-down exercise':ab,ti OR  'exergaming':ab,ti OR 'gymnastics':ab,ti OR  'physical conditioning':ab,ti OR 'endurance  training':ab,ti OR 'high-intensity interval  training':ab,ti OR 'periodization':ab,ti OR  'plyometric exercise':ab,ti OR 'resistance  training':ab,ti OR 'running':ab,ti OR  'jogging':ab,ti OR 'swimming':ab,ti OR  'walking':ab,ti OR 'stair climbing':ab,ti OR  'yoga':ab,ti OR 'tai chi':ab,ti  #4. 'exercise'/exp 553,744 25 Oct 2025  #3. #1 OR #2 66,672 25 Oct 2025  #2. 'arthroplasty, replacement, knee':ab,ti OR 53,181 25 Oct 2025  'arthroplasties, replacement, knee':ab,ti OR  'arthroplasty, knee replacement':ab,ti OR 'knee  replacement arthroplasties':ab,ti OR 'replacement  arthroplasties, knee':ab,ti OR 'knee replacement  arthroplasty':ab,ti OR 'replacement arthroplasty,  knee':ab,ti OR 'replacement, total knee':ab,ti OR  'total knee replacement':ab,ti OR 'knee  replacement, total':ab,ti OR 'knee  arthroplasty':ab,ti OR 'arthroplasty, knee':ab,ti  OR 'knee arthroplasty, total':ab,ti OR  'arthroplasties, knee replacement':ab,ti OR  'arthroplasty, total knee':ab,ti OR 'total knee  arthroplasty':ab,ti OR 'arthroplasty,  replacement, partial knee':ab,ti OR  'unicompartmental knee arthroplasty':ab,ti OR  'arthroplasty, unicompartmental knee':ab,ti OR  'knee arthroplasty, unicompartmental':ab,ti OR  'unicompartmental knee replacement':ab,ti OR  'knee replacement, unicompartmental':ab,ti OR  'partial knee replacement':ab,ti OR 'knee  replacement, partial':ab,ti OR 'unicondylar knee  replacement':ab,ti OR 'knee replacement,  unicondylar':ab,ti OR 'unicondylar knee  arthroplasty':ab,ti OR 'arthroplasty, unicondylar  knee':ab,ti OR 'knee arthroplasty,  unicondylar':ab,ti OR 'partial knee  arthroplasty':ab,ti OR 'arthroplasty, partial  knee':ab,ti OR 'knee arthroplasty, partial':ab,ti  #1. 'knee replacement'/exp 54,138 25 Oct 2025 | 2237 |
| Cochrane | ID Search Hits  #1 MeSH descriptor: [Arthroplasty, Replacement, Knee] explode all trees 4142  #2 (Arthroplasties, Replacement, Knee):ti,ab,kw or (Arthroplasty, Knee Replacement):ti,ab,kw or (Knee Replacement Arthroplasties):ti,ab,kw or (Knee Replacement Arthroplasty):ti,ab,kw or (Replacement Arthroplasties, Knee):ti,ab,kw or (Replacement Arthroplasty, Knee):ti,ab,kw or (Replacement, Total Knee):ti,ab,kw or (Total Knee Replacement):ti,ab,kw or (Knee Replacement, Total):ti,ab,kw or (Knee Arthroplasty):ti,ab,kw or (Arthroplasty, Knee):ti,ab,kw or (Arthroplasties, Knee Replacement):ti,ab,kw or (Knee Arthroplasty, Total):ti,ab,kw or (Arthroplasty, Total Knee):ti,ab,kw or (Total Knee Arthroplasty):ti,ab,kw or (Arthroplasty, Replacement, Partial Knee):ti,ab,kw or (Unicompartmental Knee Arthroplasty):ti,ab,kw or (Arthroplasty, Unicompartmental Knee):ti,ab,kw or (Knee Arthroplasty, Unicompartmental):ti,ab,kw or (Unicompartmental Knee Replacement):ti,ab,kw or (Knee Replacement, Unicompartmental):ti,ab,kw or (Partial Knee Replacement):ti,ab,kw or (Knee Replacement, Partial):ti,ab,kw or (Unicondylar Knee Replacement):ti,ab,kw or (Arthroplasty, Unicondylar Knee):ti,ab,kw or (Knee Replacement, Unicondylar):ti,ab,kw or (Unicondylar Knee Arthroplasty):ti,ab,kw or (Knee Arthroplasty, Unicondylar):ti,ab,kw or (Partial Knee Arthroplasty):ti,ab,kw or (Arthroplasty, Partial Knee):ti,ab,kw or (Knee Arthroplasty, Partial):ti,ab,kw 12022  #3 #1 or #2 12022  #4 MeSH descriptor: [Exercise] explode all trees 40925  #5 (rehabilitation):ti,ab,kw or (physiotherapy):ti,ab,kw or (Activities of daily Living):ti,ab,kw or (Early Ambulation):ti,ab,kw or (Exercise Therapy):ti,ab,kw or (Blood Flow Restriction Therapy):ti,ab,kw or (Training):ti,ab,kw or (Motion Therapy, Continuous Passive):ti,ab,kw or (Muscle Stretching Exercises):ti,ab,kw or (Telerehabilitation):ti,ab,kw or (Physical Therapy Modalities):ti,ab,kw or (Electric Stimulation Therapy ):ti,ab,kw or (Hydrotherapy ):ti,ab,kw or (Mirror Movement Therapy):ti,ab,kw or (Musculoskeletal Manipulations ):ti,ab,kw or (Myofunctional Therapy):ti,ab,kw or (Exercises):ti,ab,kw or (Physical Exercise):ti,ab,kw or (Physical Exercises):ti,ab,kw or (Exercise, Aerobic):ti,ab,kw or (Aerobic Exercise):ti,ab,kw or (Aerobic Exercises):ti,ab,kw or (Exercises, Aerobic):ti,ab,kw or (Exercise, Isometric):ti,ab,kw or (Exercises, Isometric):ti,ab,kw or (Isometric Exercises):ti,ab,kw or (Isometric Exercise):ti,ab,kw or (Acute Exercise):ti,ab,kw or (Acute Exercises):ti,ab,kw or (Exercise, Acute):ti,ab,kw or (Exercises, Acute):ti,ab,kw or (Training, Exercise):ti,ab,kw or (Trainings, Exercise):ti,ab,kw or (Physical Activity):ti,ab,kw or (Activities, Physical):ti,ab,kw or (Activity, Physical):ti,ab,kw or (Physical Activities):ti,ab,kw or (Movement):ti,ab,kw or (Motion):ti,ab,kw or (Motor Activity):ti,ab,kw or (Cool-Down Exercise):ti,ab,kw or (Exergaming):ti,ab,kw or (Gymnastics):ti,ab,kw or (Physical Conditioning):ti,ab,kw or (Endurance Training):ti,ab,kw or (High-Intensity Interval Training):ti,ab,kw or (Periodization):ti,ab,kw or (Plyometric Exercise):ti,ab,kw or (Resistance Training):ti,ab,kw or (Running):ti,ab,kw or (Jogging):ti,ab,kw or (Swimming):ti,ab,kw or (Walking):ti,ab,kw or (Stair Climbing):ti,ab,kw or (Yoga):ti,ab,kw or (Tai Chi):ti,ab,kw 379983  #6 #4 or #5 382436  #7 (Randomized controlled trials):ti,ab,kw or (placebo):ti,ab,kw or (Randomized):ti,ab,kw 1377623  #8 #3 and #6 and #7 with Publication Year from 2015 to 2025, in Trials 2629 | 2629 |
| Scopus | (( ( ( TITLE-ABS-KEY ( arthroplasty , AND replacement , AND knee ) OR TITLE-ABS-KEY ( arthroplasties , AND replacement , AND knee ) OR TITLE-ABS-KEY ( arthroplasty , AND knee AND replacement ) OR TITLE-ABS-KEY ( knee AND replacement AND arthroplasties ) OR TITLE-ABS-KEY ( knee AND replacement AND arthroplasty ) OR TITLE-ABS-KEY ( replacement AND arthroplasties , AND knee ) OR TITLE-ABS-KEY ( replacement AND arthroplasty , AND knee ) OR TITLE-ABS-KEY ( replacement , AND total AND knee ) OR TITLE-ABS-KEY ( total AND knee AND replacement ) OR TITLE-ABS-KEY ( knee AND replacement , AND total ) OR TITLE-ABS-KEY ( knee AND arthroplasty ) OR TITLE-ABS-KEY ( arthroplasty , AND knee ) OR TITLE-ABS-KEY ( arthroplasties , AND knee AND replacement ) OR TITLE-ABS-KEY ( knee AND arthroplasty , AND total ) OR TITLE-ABS-KEY ( arthroplasty , AND total AND knee ) OR TITLE-ABS-KEY ( total AND knee AND arthroplasty ) OR TITLE-ABS-KEY ( arthroplasty , AND replacement , AND partial AND knee ) OR TITLE-ABS-KEY ( unicompartmental AND knee AND arthroplasty ) OR TITLE-ABS-KEY ( arthroplasty , AND unicompartmental AND knee ) OR TITLE-ABS-KEY ( knee AND arthroplasty , AND unicompartmental ) OR TITLE-ABS-KEY ( unicompartmental AND knee AND replacement ) OR TITLE-ABS-KEY ( knee AND replacement , AND unicompartmental ) OR TITLE-ABS-KEY ( partial AND knee AND replacement ) OR TITLE-ABS-KEY ( knee AND replacement , AND partial ) OR TITLE-ABS-KEY ( unicondylar AND knee AND replacement ) OR TITLE-ABS-KEY ( knee AND replacement , AND unicondylar ) OR TITLE-ABS-KEY ( unicondylar AND knee AND arthroplasty ) OR TITLE-ABS-KEY ( arthroplasty , AND unicondylar AND knee ) OR TITLE-ABS-KEY ( knee AND arthroplasty , AND unicondylar ) OR TITLE-ABS-KEY ( partial AND knee AND arthroplasty ) OR TITLE-ABS-KEY ( arthroplasty , AND partial AND knee ) OR TITLE-ABS-KEY ( knee AND arthroplasty , AND partial ) ) ) )) AND (( TITLE-ABS-KEY ( Exercise ) OR TITLE-ABS-KEY ( rehabilitation ) OR TITLE-ABS-KEY ( physiotherapy ) OR TITLE-ABS-KEY ( Activities of daily Living ) OR TITLE-ABS-KEY ( Early Ambulation ) OR TITLE-ABS-KEY ( Exercise Therapy ) OR TITLE-ABS-KEY ( Blood Flow Restriction Therapy ) OR TITLE-ABS-KEY ( training ) OR TITLE-ABS-KEY ( Motion Therapy , Continuous Passive ) OR TITLE-ABS-KEY ( Muscle Stretching Exercises ) OR TITLE-ABS-KEY ( telerehabilitation ) OR TITLE-ABS-KEY ( Physical Therapy Modalities ) OR TITLE-ABS-KEY ( Electric Stimulation Therapy ) OR TITLE-ABS-KEY ( Hydrotherapy ) OR TITLE-ABS-KEY ( Mirror Movement Therapy ) OR TITLE-ABS-KEY ( Musculoskeletal Manipulations ) OR TITLE-ABS-KEY ( Myofunctional Therapy ) OR TITLE-ABS-KEY ( Exercises ) OR TITLE-ABS-KEY ( Physical Exercise ) OR TITLE-ABS-KEY ( Physical Exercises ) OR TITLE-ABS-KEY ( Exercise , Aerobic ) OR TITLE-ABS-KEY ( Aerobic Exercise ) OR TITLE-ABS-KEY ( Aerobic Exercises ) OR TITLE-ABS-KEY ( Exercises , Aerobic ) OR TITLE-ABS-KEY ( Exercise , Isometric ) OR TITLE-ABS-KEY ( Exercises , Isometric ) OR TITLE-ABS-KEY ( Isometric Exercises ) OR TITLE-ABS-KEY ( Isometric Exercise ) OR TITLE-ABS-KEY ( Acute Exercise ) OR TITLE-ABS-KEY ( Acute Exercises ) OR TITLE-ABS-KEY ( Exercise , Acute ) OR TITLE-ABS-KEY ( Training , Exercise ) OR TITLE-ABS-KEY ( Trainings , Exercise ) OR TITLE-ABS-KEY ( Physical Activity ) OR TITLE-ABS-KEY ( Activities , Physical ) OR TITLE-ABS-KEY ( Activity , Physical ) OR TITLE-ABS-KEY ( Physical Activities ) OR TITLE-ABS-KEY ( Movement ) OR TITLE-ABS-KEY ( Motion ) OR TITLE-ABS-KEY ( Motor Activity ) OR TITLE-ABS-KEY ( Cool-Down Exercise ) OR TITLE-ABS-KEY ( Exergaming ) OR TITLE-ABS-KEY ( Gymnastics ) OR TITLE-ABS-KEY ( Physical Conditioning ) OR TITLE-ABS-KEY ( Endurance Training ) OR TITLE-ABS-KEY ( High-Intensity Interval Training ) OR TITLE-ABS-KEY ( Periodization ) OR TITLE-ABS-KEY ( Plyometric Exercise ) OR TITLE-ABS-KEY ( Resistance Training ) OR TITLE-ABS-KEY ( running ) OR TITLE-ABS-KEY ( Jogging ) OR TITLE-ABS-KEY ( Swimming ) OR TITLE-ABS-KEY ( Walking ) OR TITLE-ABS-KEY ( Stair Climbing ) OR TITLE-ABS-KEY ( Yoga ) OR TITLE-ABS-KEY ( Tai Chi ) )) AND (( ( TITLE-ABS-KEY ( Randomized controlled trials ) OR TITLE-ABS-KEY ( Randomized ) OR TITLE-ABS-KEY ( placebo ) ) )) AND PUBYEAR > 2014 AND PUBYEAR < 2026 | 2791 |
| Web of science | 1: TS=(Arthroplasty, Knee Replacement) OR TS=(Knee Replacement Arthroplasties) OR TS=(Knee Replacement Arthroplasty) OR TS=(Replacement Arthroplasties, Knee) OR TS=(Replacement Arthroplasty, Knee) OR TS=(Replacement, Total Knee) OR TS=(Total Knee Replacement) OR TS=(Knee Replacement, Total) OR TS=(Knee Arthroplasty) OR TS=(Arthroplasty, Knee) OR TS=(Arthroplasties, Knee Replacement) OR TS=(Knee Arthroplasty, Total) OR TS=(Arthroplasty, Total Knee) OR TS=(Total Knee Arthroplasty) OR TS=(Arthroplasty, Replacement, Partial Knee) OR TS=(Unicompartmental Knee Arthroplasty) OR TS=(Arthroplasty, Unicompartmental Knee) OR TS=(Knee Arthroplasty, Unicompartmental) OR TS=(Unicompartmental Knee Replacement) OR TS=(Knee Replacement, Unicompartmental) OR TS=(Partial Knee Replacement) OR TS=(Knee Replacement, Partial) OR TS=(Unicondylar Knee Replacement) OR TS=(Knee Replacement, Unicondylar) OR TS=(Unicondylar Knee Arthroplasty) OR TS=(Arthroplasty, Unicondylar Knee) OR TS=(Knee Arthroplasty, Unicondylar) OR TS=(Partial Knee Arthroplasty) OR TS=(Arthroplasty, Partial Knee) OR TS=(Knee Arthroplasty, Partial) OR TS=(Arthroplasty, Replacement, Knee) OR TS=(Arthroplasties, Replacement, Knee) Date Run: Sun Oct 26 2025 11:25:40 GMT+0800 Results: 65517  2: TS=(Exercise) OR TS=(rehabilitation) OR TS=(physiotherapy) OR TS=(Activities of daily Living) OR TS=(Early Ambulation) OR TS=(Exercise Therapy) OR TS=(Blood Flow Restriction Therapy) OR TS=(Training) OR TS=(Motion Therapy, Continuous Passive) OR TS=(Telerehabilitation) OR TS=(Muscle Stretching Exercises) OR TS=(Physical Therapy Modalities) OR TS=(Electric Stimulation Therapy ) OR TS=(Hydrotherapy ) OR TS=(Mirror Movement Therapy) OR TS=(Musculoskeletal Manipulations ) OR TS=(Myofunctional Therapy) OR TS=(Exercises) OR TS=(Physical Exercise) OR TS=(Physical Exercises) OR TS=(Exercise, Aerobic) OR TS=(Aerobic Exercise) OR TS=(Aerobic Exercises) OR TS=(Exercises, Aerobic) OR TS=(Exercises, Isometric) OR TS=(Exercise, Isometric) OR TS=(Isometric Exercises) OR TS=(Isometric Exercise) OR TS=(Acute Exercise) OR TS=(Acute Exercises) OR TS=(Exercise, Acute) OR TS=(Exercises, Acute) OR TS=(Training, Exercise) OR TS=(Trainings, Exercise) OR TS=(Physical Activity) OR TS=(Activities, Physical) OR TS=(Activity, Physical) OR TS=(Physical Activities) OR TS=(Movement) OR TS=(Motion) OR TS=(Motor Activity) OR TS=(Cool-Down Exercise) OR TS=(Exergaming) OR TS=(Gymnastics) OR TS=(Physical Conditioning) OR TS=(Endurance Training) OR TS=(High-Intensity Interval Training) OR TS=(Periodization) OR TS=(Plyometric Exercise) OR TS=(Resistance Training) OR TS=(Running) OR TS=(Jogging) OR TS=(Swimming) OR TS=(Walking) OR TS=(Stair Climbing) OR TS=(Yoga) OR TS=(Tai Chi) Date Run: Sun Oct 26 2025 11:26:05 GMT+0800 Results: 5959746  3: ((AB=(Randomized controlled trials)) OR AB=(Randomized)) OR AB=(placebo) Date Run: Sun Oct 26 2025 11:26:25 GMT+0800 Results: 977502  4: #1 AND #2 AND #3 Date Run: Sun Oct 26 2025 11:27:01 GMT+0800 Results: 2811  5: #1 AND #2 AND #3 and 2015 or 2016 or 2017 or 2018 or 2019 or 2020 or 2021 or 2024 or 2025 or 2022 or 2023 (Publication Years) Date Run: Sun Oct 26 2025 11:27:18 GMT+0800 Results: 2082 | 2082 |

# Supplementary Information S3. Included types of exercise, definitions, and core content

The nine interventions included in this network meta-analysis were grouped based on the clinical decision-making scenarios encountered in early rehabilitation after total knee arthroplasty. Specifically:

## Table 3 Included types of exercise, definitions, and core content

| **Secondary Category (Intervention)** | **Definition and Core Components** |
| --- | --- |
| **1. Conventional Physical Therapy (CPT)** (Studies n=26, Patients n=732) | **Definition**: Served as the control benchmark. Refers to a comprehensive exercise therapy protocol led by a physical therapist, following standard hospital or clinic procedures. **Core Components**: Basic strength training (e.g., straight leg raise), range of motion exercises, balance training, gait training. |
| **2. Active Range of Motion Training (AROM-T)** (Studies n=4, Patients n=145) | **Definition**: A training protocol centered on a limited number of active range of motion exercises, standardized and managed without reliance on complex equipment. **Core Components**: Standardized active heel slides, isometric quadriceps training, etc. |
| **3. Resistance Training (RT)** (Studies n=2, Patients n=53) | **Definition**: A structured and progressive training protocol where external resistance is a core element, implemented according to specific parameters. **Core Components**: Lower limb resistance exercises using elastic bands, sandbags, weight machines, etc. |
| **4. Functional Training (FT)** (Studies n=6, Patients n=142) | **Definition**: A task-oriented exercise therapy focusing on simulating and training movement patterns required for activities of daily living. **Core Components**: Sit-to-stand transfers, stair ascent/descent, squats, lunges, and proprioceptive, balance, and walking training on complex surfaces. |
| **5. Continuous Passive Motion (CPM)** (Studies n=11, Patients n=446) | **Definition**: The use of a dedicated CPM machine to provide fully passive, continuous range of motion exercise in the early postoperative period. **Core Components**: Any study where CPM was the primary or sole exercise intervention. |
| **6. Device-Assisted Active Training (DAAT)** (Studies n=5, Patients n=144) | **Definition**: An active exercise therapy whose core relies on specialized physical devices to guide, control, quantify, or provide biofeedback during movement. **Core Components**: Controlled active motion (e.g., CAM), single-joint hybrid assistive braces, etc. |
| **7. Intelligence-Assisted Training (IAT)** (Studies n=6, Patients n=143) | **Definition**: An exercise therapy whose core relies on technologies such as Virtual Reality (VR), Augmented Reality (AR), or mobile applications (Apps) to create a digital, interactive training environment. **Core Components**: Gamified training using VR/AR headsets; or standardized training sessions guided by video instructions via a mobile App. |
| **8. ERAS-based rehabilitation pathway (ERAS)** (Studies n=3, Patients n=108) | **Definition**: A multimodal, structured perioperative exercise protocol developed under the guidance of the Enhanced Recovery After Surgery (ERAS) concept. **Core Components**: Preoperative prehabilitation, very early postoperative ambulation, goal-directed daily quantitative functional training, and high-intensity progressive rehabilitation protocols. |
| **9. Motor Imagery (MI)** (Studies n=3, Patients n=38) | **Definition**: A cognitive neurorehabilitation technique where patients consciously simulate and rehearse a movement in their mind without muscular activation. **Core Components**: Therapist-guided, systematic imagination of activities such as knee flexion/extension, walking, etc. |

# Supplementary Information S4. The list for the included studies

1. Jin C, Feng YJ, Ni YJ, Shan ZL. Virtual reality intervention in postoperative rehabilitation after total knee arthroplasty: a prospective and randomized controlled clinical trial. Int J Clin Exp Med. 2018;11(6):6119-24.
2. Maeda T, Sasaki E, Kasai T, Igarashi S, Wakai Y, Sasaki T, et al. Therapeutic effect of knee extension exercise with single-joint hybrid assistive limb following total knee arthroplasty: a prospective, randomized controlled trial. Sci Rep. 2024;14(1):3889-990.
3. Stasi S, Baltopoulos IP, Papathanasiou G, Korres NI. The efficacy of continuous passive motion after total knee arthroplasty a three-group randomized controlled trial. Arch Hell Med. 2020;37(3):341-53.
4. Lee H-G, An J, Lee B-H. The Effect of Progressive Dynamic Balance Training on Physical Function, The Ability to Balance and Quality of Life Among Elderly Women Who Underwent a Total Knee Arthroplasty: A Double-Blind Randomized Control Trial. Int J Environ Res Public Health. 2021;18(5):2513-23.
5. Candiri B, Talu B, Guner E, Ozen M. The effect of graded motor imagery training on pain, functional performance, motor imagery skills, and kinesiophobia after total knee arthroplasty: randomized controlled trial. Korean J Pain. 2023;36(3):369-81.
6. Schulz M, Krohne B, Röder W, Sander K. Randomized, prospective, monocentric study to compare the outcome of continuous passive motion and controlled active motion after total knee arthroplasty. Technol Health Care. 2018;26(3):499-506.
7. Shim GY, Kim EH, Lee SJ, Chang CB, Lee YS, Lee JI, et al. Postoperative rehabilitation using a digital healthcare system in patients with total knee arthroplasty: a randomized controlled trial. Arch Orthop Trauma Surg. 2023;143(10):6361-70.
8. Hardt S, Schulz MRG, Pfitzner T, Wassilew G, Horstmann H, Liodakis E, et al. Improved early outcome after TKA through an app-based active muscle training programme—a randomized-controlled trial. Knee Surg Sports Traumatol Arthrosc. 2018;26(11):3429-37.
9. Wirries N, Ezechieli M, Stimpel K, Skutek M. Impact of continuous passive motion on rehabilitation following total knee arthroplasty. Physiother Res Int. 2020;25(4):e1869.
10. Paravlic AH, Maffulli N, Kovač S, Pisot R. Home-based motor imagery intervention improves functional performance following total knee arthroplasty in the short term: a randomized controlled trial. J Orthop Surg Res. 2020;15(1):451-66.
11. Jiao S, Feng Z, Dai T, Huang J, Liu R, Meng Q. High-Intensity Progressive Rehabilitation Versus Routine Rehabilitation After Total Knee Arthroplasty: A Randomized Controlled Trial. J Arthroplasty. 2024;39(3):665-71.e2.
12. Jiao S, Feng Z, Huang J, Dai T, Liu R, Meng Q. Enhanced recovery after surgery combined with quantitative rehabilitation training in early rehabilitation after total knee replacement: a randomized controlled trial. Eur J Phys Rehabil Med. 2024;60(1):74-83.
13. Nishitha K, Anitha A, Thaheera D. Effectiveness of Virtual Reality-based Rehabilitation and High-intensity Exercise Program for Total Knee Arthroplasty Patients: A Randomised Controlled Trial. J Clin Diagn Res. 2024;18(11):YC1-YC8.
14. Hasubhai PZ, D BD, A TR. Effectiveness of Conventional Physiotherapy along with Continuous Passive Motion after Total Knee Arthroplasty. Indian J Physiother Occup Ther. 2017;11(4):195‐200.
15. Sahni G, Singh S, Singh D, Kavia A. Effectiveness of continuous passive motion after total knee replacement: A randomized controlled trial from North India. Natl J Physiol Pharm Pharmacol. 2022;12(10):1556-9.
16. Baloch N, Zubairi AJ, Rashid RH, Hashmi PM, Lakdawala RH. Effect of continuous passive motion on knee flexion range of motion after total knee arthroplasty. J Pak Med Assoc. 2015;65(11 Suppl 3):S32-4.
17. An J, Son Y-W, Lee B-H. Effect of Combined Kinematic Chain Exercise on Physical Function, Balance Ability, and Gait in Patients with Total Knee Arthroplasty: A Single-Blind Randomized Controlled Trial. Int J Environ Res Public Health. 2023;20(4):3524-37.
18. Shabbir M, Umar B, Ehsan S, Munir S, Bunin U, Sarfraz K. Comparison of functional training and strength training in improving knee extension lag after first four weeks of total knee replacement. Biomed Res-India. 2017;28(12):5623-7.
19. Eymir M, Erduran M, Ünver B. Active heel‐slide exercise therapy facilitates the functional and proprioceptive enhancement following total knee arthroplasty compared to continuous passive motion. Knee Surg Sports Traumatol Arthrosc. 2020;29(10):3352-60.
20. Yasaci Z, Celik D, Kilicoglu OI. Short-Term Efficacy of Integrating the Easy-Flex Device With Inpatient Rehabilitation in Individuals With Total Knee Arthroplasty: Insights From a Single-Blind Randomized Controlled Trial. Arch Phys Med Rehabil. 2024;105(11):2045-53.
21. An J, Cheon S-J, Lee B-H. The Effect of Combined Balance Exercise on Knee Range of Motion, Balance, Gait, and Functional Outcomes in Acute Phase Following Total Knee Arthroplasty: A Single-Blind Randomized Controlled Trial. Medicina. 2024;60(9):1389-401.
22. Núñez-Cortés R, López-Bueno L, López-Bueno R, Cuenca-Martínez F, Suso-Martí L, Silvestre A, et al. Acute Effects of In-Hospital Resistance Training on Clinical Outcomes in Patients Undergoing Total Knee Arthroplasty. Am J Phys Med Rehabil. 2024;103(5):401-9.
23. Goetz J, Maderbacher G, Gerg A, Leiss F, Dullien S, Zeman F, et al. Isokinetic knee muscle strength comparison after enhanced recovery after surgery (ERAS) versus conventional setup in total knee arthroplasty (TKA): a single blinded prospective randomized study. J Exp Orthop. 2023;10(1):44-56.
24. Yu J-H, Nekar DM, Kang H-Y, Lee J-W, Oh S-Y. Comparison of Physical Activity Training Using Augmented Reality and Conventional Therapy on Physical Performance following a Total Knee Replacement: A Randomized Controlled Trial. Appl Sci. 2023;13(2):894-905.
25. Arslan HM, Riaz S, Ashraf A, Hashmi Z, Khan RR, Rashid S. Effectiveness of Structured Rehabilitation Program on Pain and Function in Patients with Total Knee Replacement: A Randomized Controlled Trial. Pak J Med Health Sci. 2022;16(1):1376-9.
26. Cook JL, Rucinski K, Crecelius CR, Cunningham S, Guess TM. Prospective, Randomized Clinical Trial Comparing a Novel Motion-Assistive Device to Standard Physical Therapy for Initial Management of Knee Range of Motion after Primary Total Knee Arthroplasty. J Knee Surg. 2021;36(04):424-30.
27. Bäcker HC, Wu CH, Schulz MRG, Weber-Spickschen TS, Perka C, Hardt S. App-based rehabilitation program after total knee arthroplasty: a randomized controlled trial. Arch Orthop Trauma Surg. 2021;141(9):1575-82.
28. Joshi RN, White PB, Murray-Weir M, Alexiades MM, Sculco TP, Ranawat AS. Prospective Randomized Trial of the Efficacy of Continuous Passive Motion Post Total Knee Arthroplasty: Experience of the Hospital for Special Surgery. J Arthroplasty. 2015;30(12):2364-9.
29. Kondo Y, Yoshida Y, Iioka T, Kataoka H, Sakamoto J, Hirase T, et al. Short-Term Effects of Isometric Quadriceps Muscle Exercise with Auditory and Visual Feedback on Pain, Physical Function, and Performance after Total Knee Arthroplasty: A Randomized Controlled Trial. J Knee Surg. 2020;35(08):922-31.
30. Fitz W, Shukla P, Li L, Scott RD. Early Regain of Function and Proprioceptive Improvement Following Knee Arthroplasty. Arch Bone Jt Surg. 2018;6:523-31.
31. Gil-González S, Barja-Rodríguez RA, López-Pujol A, Berjaoui H, Fernández-Bengoa JE, Erquicia JI, et al. Continuous passive motion not affect the knee motion and the surgical wound aspect after total knee arthroplasty. J Orthop Surg Res. 2022;17(1):25-32.
32. Briones-Cantero M, Fernández-de-las-Peñas C, Lluch-Girbés E, Osuna-Pérez MC, Navarro-Santana MJ, Plaza-Manzano G, et al. Effects of Adding Motor Imagery to Early Physical Therapy in Patients with Knee Osteoarthritis who Had Received Total Knee Arthroplasty: A Randomized Clinical Trial. Pain Med. 2020;21(12):3548-55.

# Supplementary Information S5. Risk of bias of included studies

The risk of bias and quality of the included studies were independently assessed by at least two reviewers using the revised Cochrane risk-of-bias tool for randomized trials (RoB2). This tool evaluates five domains of potential bias in randomized controlled trials: the randomization process, deviations from intended interventions, missing outcome data, measurement of the outcome, and selection of the reported result. Each domain consists of a series of questions with response options (“low,” “some concerns,” or “high”) used to classify the risk-of-bias judgment. For the overall risk-of-bias rating, studies were categorized as “low risk,” “some concerns,” or “high risk.” An overall judgment of “low risk of bias” was assigned if all five domains were rated as low risk; “some concerns” if at least one domain was judged as having some concerns and no domain was rated as high risk; and “high risk of bias” if at least one domain was rated as high risk. Any discrepancies between the reviewers’ assessments were resolved through discussion with a third reviewer.

**1. Bias Arising from the Randomization Process**

1. Low Risk: Random sequence generation and allocation concealment are appropriately implemented, and no significant baseline imbalances are present
2. Some Concerns: There is insufficient information about the randomization or allocation concealment process, or minor imbalances exist between intervention groups
3. High Risk: Randomization is not properly conducted, allocation concealment is inadequate, or significant baseline imbalances indicate manipulation

**2. Bias Due to Deviations from Intended Interventions**

1. Low Risk: The trial follows the protocol without significant deviations, and participants adhere to the assigned intervention.
2. Some Concerns: There are minor deviations or non-adherence that might affect outcomes but are not expected to introduce major bias.
3. High Risk: Significant deviations occur, such as unplanned interventions or lack of blinding, that could influence the trial's results.

**3. Bias Due to Missing Outcome Data**

1. Low Risk: Missing data are minimal and unlikely to impact the results.
2. Some Concerns: Some missing data could affect outcomes but do not critically threaten validity.
3. High Risk: Large amounts of missing data substantially reduce the reliability of the results.

**4. Bias in Measurement of the Outcome**

1. Low Risk: Outcomes are measured using validated and reliable methods with minimal risk of bias.
2. Some Concerns: Measurement tools or procedures may introduce some uncertainty, but the impact on the outcome is limited.
3. High Risk: Inappropriate measurement methods or lack of blinding lead to biased outcome assessment.

**5. Bias in Selection of the Reported Result**

1. Low Risk: Reported results align with pre-specified outcomes without evidence of selective reporting.
2. Some Concerns: There are slight discrepancies between reported and pre-specified outcomes, but they do not heavily impact conclusions.
3. High Risk: Selective reporting is evident, with only favorable outcomes reported, significantly affecting the validity of the study.

**6. Overall Risk-of-Bias Judgement**

1. Low Risk-of-Bias: The trial is judged to be at low risk of bias for all domains for this result.
2. Some Concerns: The trial is judged to raise some concerns in one to three domains but is not at high risk of bias in any.
3. High Risk-of-Bias: The trial is judged to be at high risk of bias in at least one domain for this result, OR the trial is judged to have some concerns in more than three domains, significantly reducing confidence in the result.

## Table 4 Quality evaluation of the included literature

| Study | Randomization process | Deviations from intended interventions | Missing outcomes data | Measurement of the outcome | Selection of the reported result | overall Bias |
| --- | --- | --- | --- | --- | --- | --- |
| Jin et al. 2018 | Some concerns | Low | Low | High | Low | High |
| Maeda et al. 2024 | Some concerns | Low | Low | Low | Low | Some concerns |
| Stasi et al. 2020 | Some concerns | Low | Low | Low | Low | Some concerns |
| Lee et al. 2021 | Some concerns | Low | Low | Low | Some concerns | Some concerns |
| Candiri et al. 2023 | Low | High | Low | Low | Low | High |
| Schulz et al. 2018 | Low | Low | Low | Low | Low | Low |
| Shim et al. 2023 | Low | Low | Low | Low | Low | Low |
| Hardt et al. 2018 | Low | Some concerns | Low | High | Low | High |
| Wirries et al. 2020 | Low | Some concerns | Low | High | Low | High |
| Paravlic et al. 2020 | Low | Some concerns | Low | Low | Low | Some concerns |
| Jiao et al. 2024 | Low | Low | Low | Low | Low | Low |
| Jiao et al. 2024 | Low | Low | Low | Low | Low | Low |
| Briones-Cantero et al. 2020 | Low | Low | Low | Low | Low | Low |
| Nishitha et al. 2024 | Some concerns | Some concerns | Low | Low | Low | Some concerns |
| Patel et al. 2017 | Some concerns | Some concerns | Low | Low | Some concerns | Some concerns |
| Sahni et al. 2022 | Some concerns | Low | Low | Low | Some concerns | Some concerns |
| Baloch et al. 2015 | Low | Low | Low | Low | Some concerns | Some concerns |
| An et al. 2023 | Some concerns | Some concerns | Low | Low | Some concerns | Some concerns |
| Shabbir et al. 2017 | Low | Low | Low | Low | Low | Low |
| Eymir et al. 2020 | Some concerns | Some concerns | Low | High | Some concerns | High |
| Yasaci et al. 2024 | Low | Some concerns | Low | Low | Low | Some concerns |
| An et al. 2024 | Some concerns | Some concerns | Low | Low | Low | Some concerns |
| Núñez-Cortés et al. 2024 | Low | Low | Low | High | Low | High |
| Goetz et al. 2023 | Low | Some concerns | Low | High | Low | High |
| Yu et al. 2023 | Low | Some concerns | Low | Low | Low | Some concerns |
| Arslan et al. 2022 | Some concerns | Some concerns | Low | High | Some concerns | High |
| Cook et al. 2021 | Low | Some concerns | Low | Low | Some concerns | Some concerns |
| Bäcker et al. 2021 | Low | Some concerns | Low | High | Some concerns | High |
| Joshi et al. 2015 | Low | Some concerns | Low | Low | Some concerns | Some concerns |
| Kondo et al. 2020 | Some concerns | Some concerns | Low | Low | Some concerns | Some concerns |
| Fitz et al. 2018 | Low | Low | Low | Low | Low | Low |
| Gil-González et al. 2022 | Some concerns | Low | Low | Low | Some concerns | Some concerns |
| Total | | | | | | |
| Low n (%) | 19(59.38%） | 15(46.88%) | 32(100%) | 24(75%) | 19(59.38%) | 7(21.88%) |
| Some concerns n (%) | 13(40.62%） | 16(50%) | 0 | 0 | 13(40.62%) | 16(50%) |
| high n (%) | 0 | 1(3.12%) | 0 | 8(25%) | 0 | 9(28.12%) |

# Supplementary Information S6. The analysis for primary outcomes of pain

**1.Inconsistency of different intervention**

**（1）Global inconsistency test**

| Inconsistency | chi^2^ | Prob>chi^2^ |
| --- | --- | --- |
| design-by-treatment | 1.54 | 0.9088 |

**（2）Part of side splitting inconsistency**

| Side | P>z | tau | Treatments | |
| --- | --- | --- | --- | --- |
| A B | 0.447 | 0.3180116 | A: | CPT |
| A C | 0.987 | 0.3365444 | B: | IAT |
| A D | - | - | C: | CPM |
| A E | - | - | D: | MI |
| A F | 0.942 | 0.3235196 | E: | ERAS |
| A G | 0.939 | 0.3235196 | F: | RT |
| A H | 0.931 | 0.3354092 | G: | FT |
| A I | 0.548 | 0.3267906 | H: | DAAT |
| B C | 0.447 | 0.3180133 | I: | AROM-T |
| C H | 0.581 | 0.3270981 |  |  |
| C I | 0.190 | 0.2909698 |  |  |
| F G | 0.940 | 0.3235229 |  |  |
| H I | 0.502 | 0.32479 |  |  |

*Abbreviations.* A: CPT, Conventional Physical Therapy；B: IAT, Intelligence-Assisted Training；C: CPM, Continuous Passive Motion；D: MI, Motor Imagery；E: ERAS, ERAS-based rehabilitation pathway；F: RT, Resistance Training；G: FT, Functional Training；H: DAAT, Device-Assisted Active Training；I: AROM-T, Active Range of Motion Training；

**2. Local inconsistency test**

**
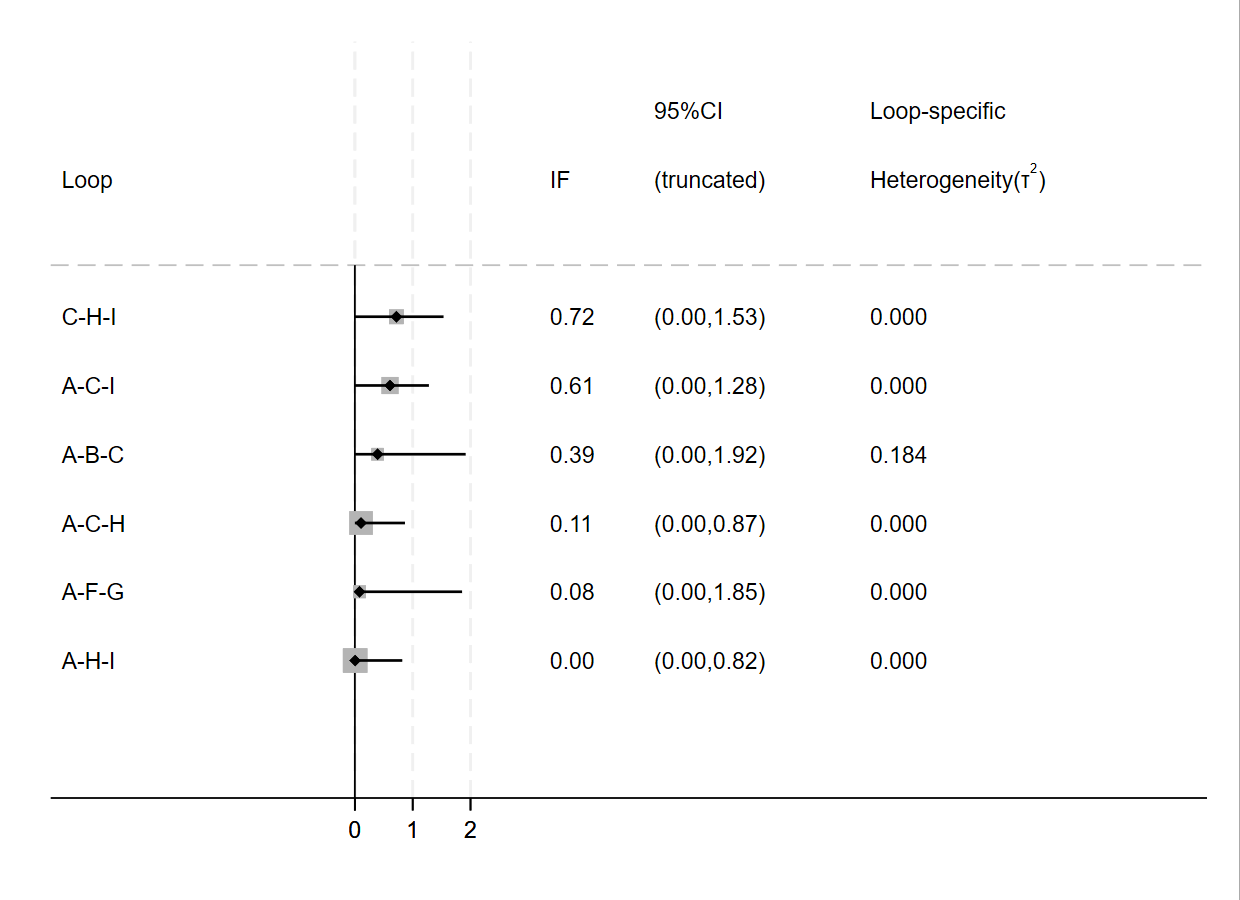
**

## Figure 1 Loop-specific heterogeneity for pain

*Note.* A: CPT, Conventional Physical Therapy；B: IAT, Intelligence-Assisted Training；C: CPM, Continuous Passive Motion；D: MI, Motor Imagery；E: ERAS, ERAS-based rehabilitation pathway；F: RT, Resistance Training；G: FT, Functional Training；H: DAAT, Device-Assisted Active Training；I: AROM-T, Active Range of Motion Training；

1. **Forest Plot of Pain Outcome Indicators**

**
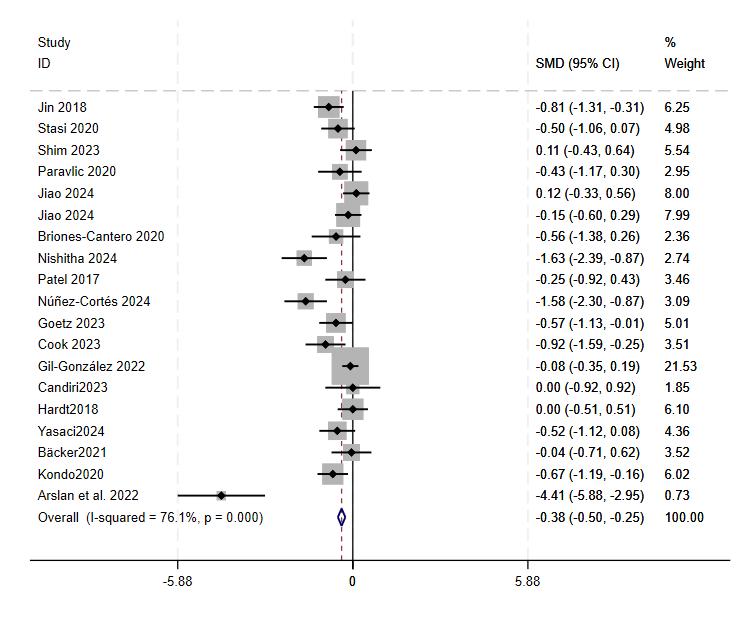
**

## Figure 2 Forest plot of pain outcome indicators

**4.The contributions of direct and indirect estimates for the overall estimates of effect sizes**


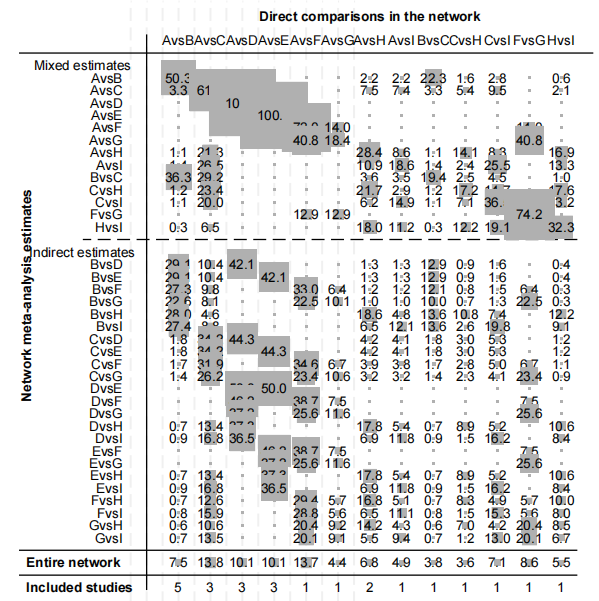


## Figure 3 Contribution plot for pain

*Note*. A: CPT, Conventional Physical Therapy；B: IAT, Intelligence-Assisted Training；C: CPM, Continuous Passive Motion；D: MI, Motor Imagery；E: ERAS, ERAS-based rehabilitation pathway；F: RT, Resistance Training；G: FT, Functional Training；H: DAAT, Device-Assisted Active Training；I: AROM-T, Active Range of Motion Training；

1. **Funnel plot for publication bias testing in the included studies**

**
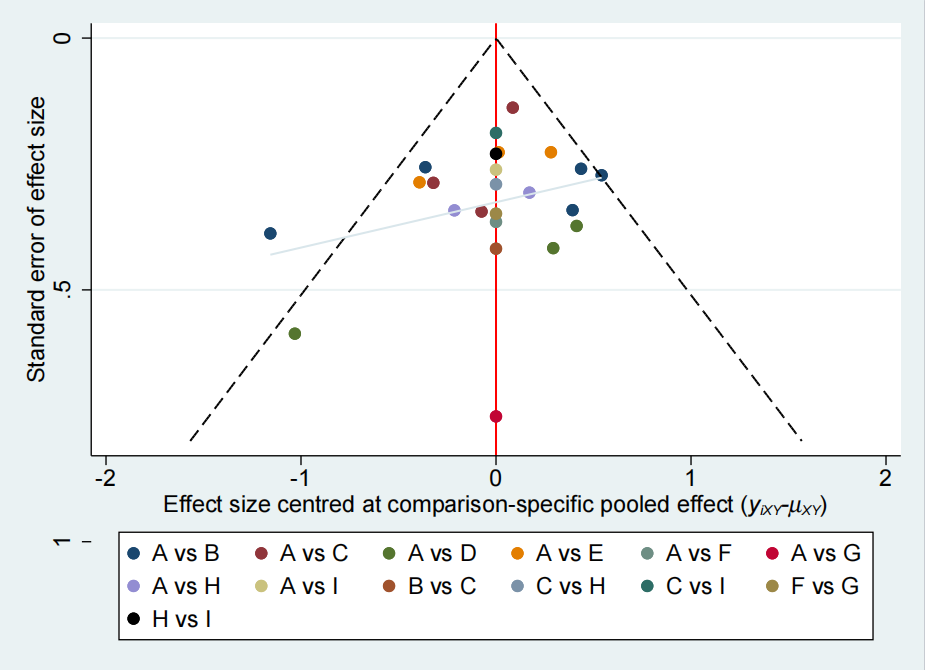
**

## Figure 4 Funnel plot of pain outcome measures

*Note*. A: CPT, Conventional Physical Therapy；B: IAT, Intelligence-Assisted Training；C: CPM, Continuous Passive Motion；D: MI, Motor Imagery；E: ERAS, ERAS-based rehabilitation pathway；F: RT, Resistance Training；G: FT, Functional Training；H: DAAT, Device-Assisted Active Training；I: AROM-T, Active Range of Motion Training；

# Supplementary Information S7. The analysis for primary outcomes of WOMAC

**1.Inconsistency of different intervention**

1. **Global inconsistency test**

| Inconsistency | chi^2^ | Prob>chi^2^ |
| --- | --- | --- |
| design-by-treatment | 1.94 | 0.3796 |

**（2）Part of side splitting inconsistency**

| Side | P>z | tau | Treatments | |
| --- | --- | --- | --- | --- |
| A B | - | - | A: | CPT |
| A C | 0.301 | 0.6676055 | B: | IAT |
| A D | - | - | C: | FT |
| A E | 0.350 | 0.6773216 | D: | MI |
| A F | 0.350 | 0.6773424 | E: | CPM |
| A G | - | - | F: | DAAT |
| A H | 0.300 | 0.6676254 | G: | RT |
| C H | 0.300 | 0.667633 | H: | AROM-T |
| E F | 0.350 | 0.6773501 |  |  |

*Note.* A: CPT, Conventional Physical Therapy；B: IAT, Intelligence-Assisted Training;；C: FT：Functional Training; D: MI：Motor Imagery；E: CPM:Continuous Passive Motion；F: DAAT：Device-Assisted Active Training; G: RT：Resistance Training；H: AROM-T：Active Range of Motion Training；

**2. Local inconsistency test**

**
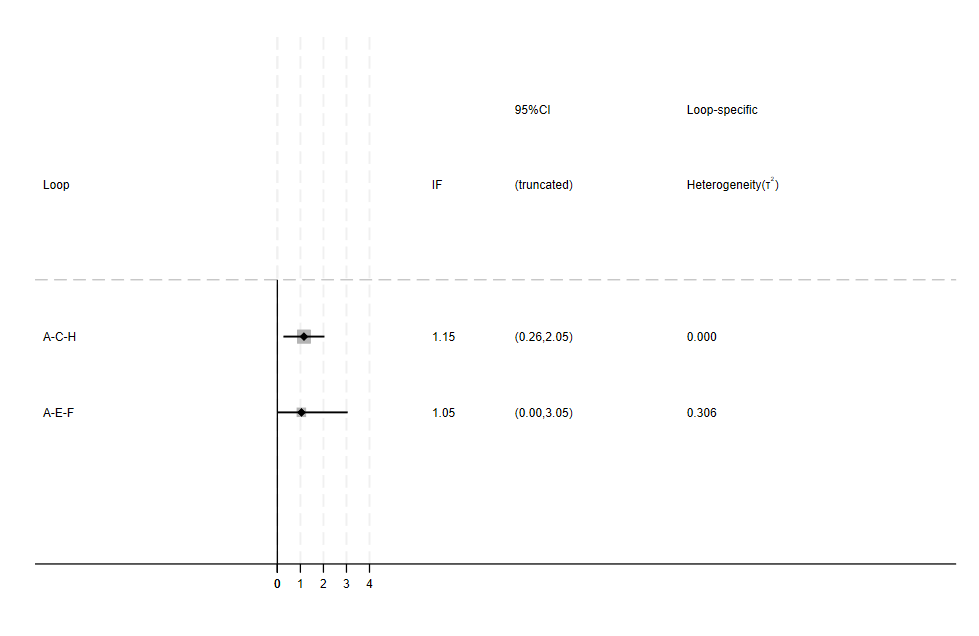
**

## Figure 5 Loop-specific heterogeneity for WOMAC

*Note.* A: CPT, Conventional Physical Therapy；C:FT：Functional Training; E: CPM, Continuous Passive Motion；F:DAAT：Device-Assisted Active Training; H:AROM-T：Active Range of Motion Training；

**3.Forest Plot of WOMAC Outcome Indicators**

**
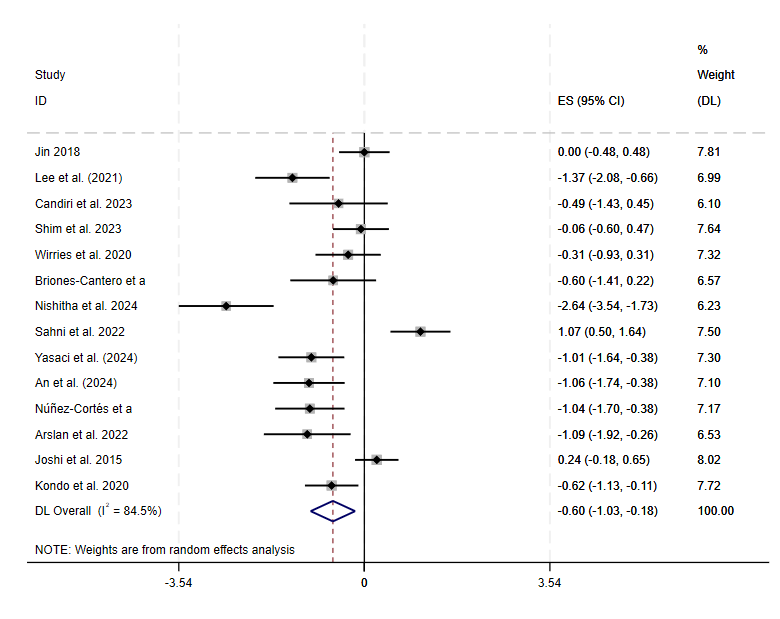
**

## Figure 6 Forest Plot of WOMAC Outcome Indicators

**4.The contributions of direct and indirect estimates for the overall estimates of effect sizes**


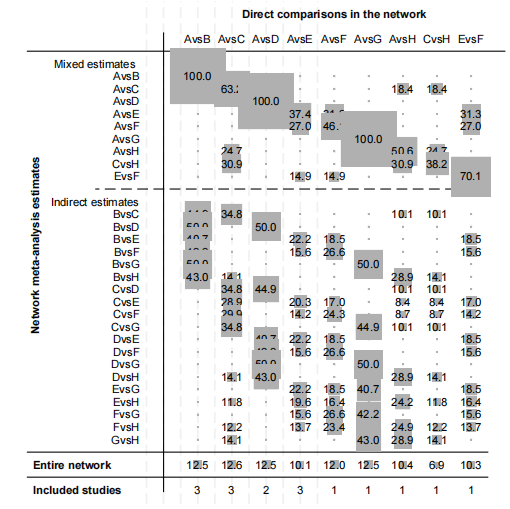


## Figure 7 Contribution plot for WOMAC

*Note.* A: CPT, Conventional Physical Therapy；B: IAT, Intelligence-Assisted Training；C:FT：Functional Training; D: MI, Motor Imagery；E: CPM, Continuous Passive Motion；F:DAAT：Device-Assisted Active Training; G:RT：Resistance Training；H:AROM-T：Active Range of Motion Training；

**4.Funnel plot for publication bias testing in the included studies**

**
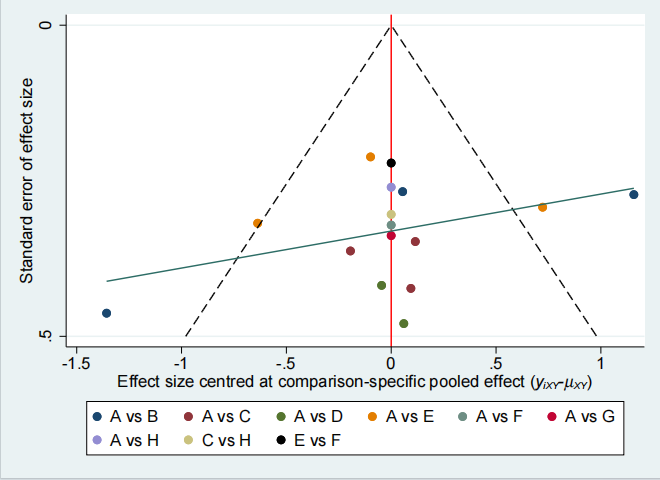
**

## Figure 8 Funnel plot of WOMAC outcome measures

*Note.* A: CPT, Conventional Physical Therapy；B: IAT, Intelligence-Assisted Training；C:FT：Functional Training; D: MI, Motor Imagery；E: CPM, Continuous Passive Motion；F:DAAT：Device-Assisted Active Training; G:RT：Resistance Training；H:AROM-T：Active Range of Motion Training；

# Supplementary Information S8. The analysis for primary outcomes of ROM

**1.Inconsistency of different intervention**

**（1）Global inconsistency test**

| Inconsistency | chi^2^ | Prob>chi^2^ |
| --- | --- | --- |
| design-by-treatment | 1.78 | 0.6201 |

**（2）Part of side splitting inconsistency**

| Side | P>z | tau | Treatments | |
| --- | --- | --- | --- | --- |
| A B | 0.847 | 0.5295145 | A: | CPT |
| A C | - | - | B: | CPM |
| A D | - | - | C: | FT |
| A E | 0.979 | 0.530616 | D: | MI |
| A F | 0.194 | 0.4754742 | E: | DAAT |
| A G | - | - | F: | IAT |
| A H | 0.501 | 0.5201033 | G: | RT |
| B E | 0.979 | 0.5306181 | H: | AROM-T |
| B F | 0.195 | 0.475476 |  |  |
| B H | 0.501 | 0.5201065 |  |  |

*Abbreviations.* A: CPT, Conventional Physical Therapy；B:CPM:Continuous Passive Motion；C:FT：Functional Training; D: MI, Motor Imagery；E:DAAT：Device-Assisted Active Training; F:IAT：Intelligence-Assisted Training；G:RT：Resistance Training；H:AROM-T：Active Range of Motion Training；

**2. Local inconsistency test**

**
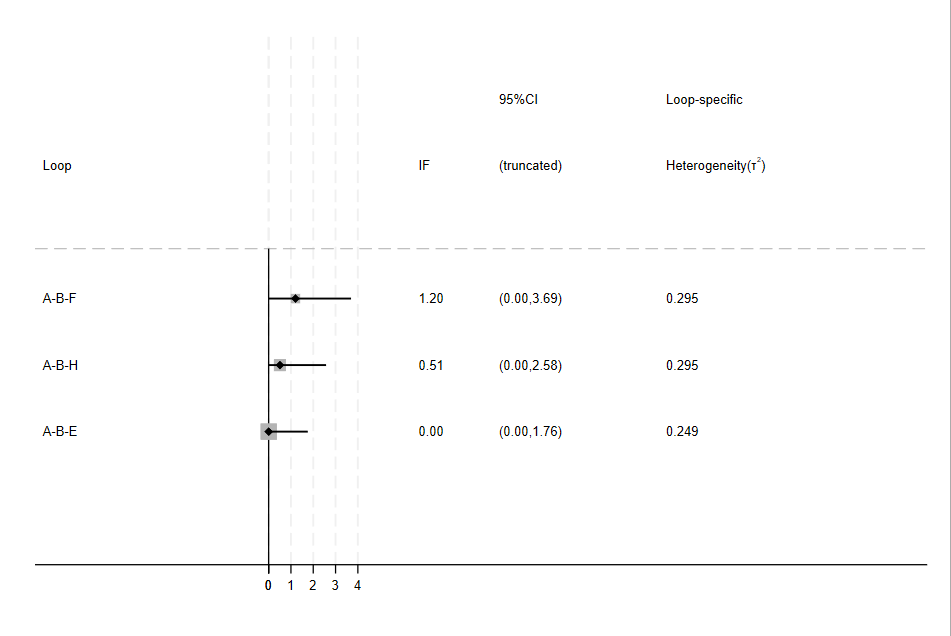
**

## Figure 9 Loop-specific heterogeneity for ROM

*Note:* A: CPT, Conventional Physical Therapy；B:CPM:Continuous Passive Motion；E:DAAT：Device-Assisted Active Training; F:IAT：Intelligence-Assisted Training；H:AROM-T：Active Range of Motion Training；

**3.Forest Plot of ROM Outcome Indicators**

**
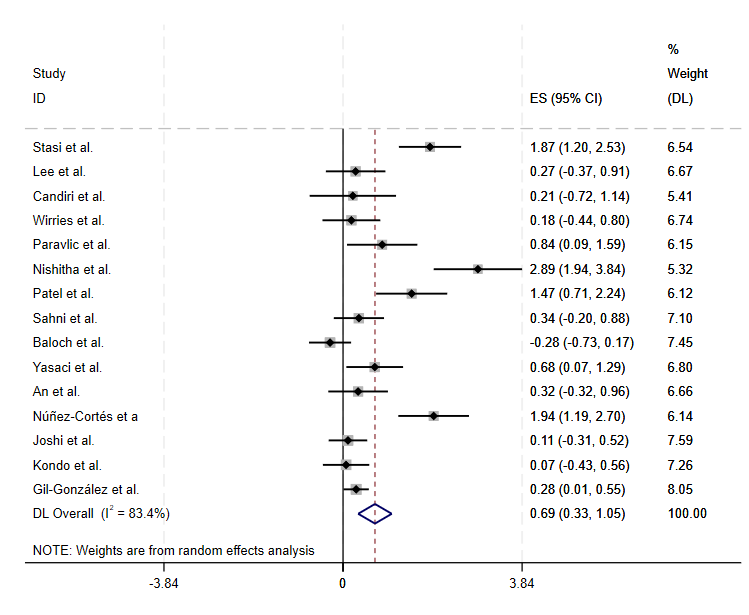
**

## Figure 10 Forest Plot of ROM Outcome Indicators

**4.The contributions of direct and indirect estimates for the overall estimates of effect sizes**


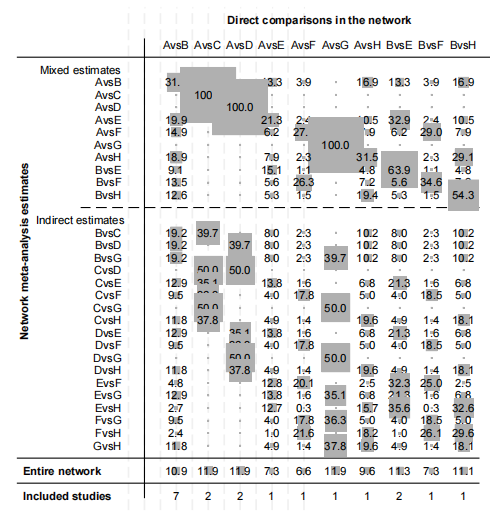


## Figure 11 Contribution plot for ROM

*Note*. A: CPT, Conventional Physical Therapy；B: CPM:Continuous Passive Motion；C:FT：Functional Training; D: MI, Motor Imagery；E:DAAT：Device-Assisted Active Training; F:IAT：Intelligence-Assisted Training；G:RT：Resistance Training；H:AROM-T：Active Range of Motion Training；

**4.Funnel plot for publication bias testing in the included studies**

**
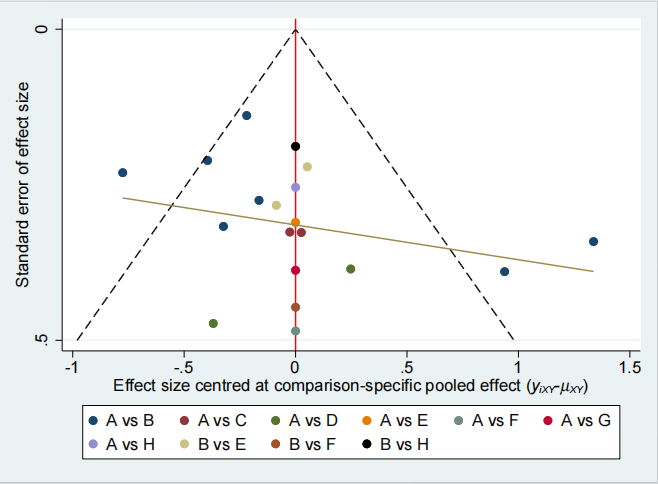
**

## Figure 12 Funnel plot of ROM outcome measures

*Note*. A: CPT, Conventional Physical Therapy；B: CPM, Continuous Passive Motion；C: FT, Functional Training; D: MI, Motor Imagery；E: DAAT, Device-Assisted Active Training; F: IAT, Intelligence-Assisted Training；G: RT, Resistance Training；H: AROM-T, Active Range of Motion Training；

# Supplementary Information S9. Assessment of evidence certainty

## Table 5 CINeMA Assessment of evidence certainty

| Outcome | Risk of Bias | Publication Bias | Indirectness | Imprecision | **Heterogeneity** | Inconsistency | Quality of Evidence |
| --- | --- | --- | --- | --- | --- | --- | --- |
| pain | -1 | 0 | 0 | 0 | -1 | 0 | Moderate |
| WOMAC | -1 | 0 | -1 | 0 | -1 | 0 | Low |
| ROM | -1 | 0 | -1 | 0 | -1 | 0 | Low |
